# An ER-Associated Pathway Defines Endosomal Architecture for Controlled Cargo Transport

**DOI:** 10.1016/j.cell.2016.05.078

**Published:** 2016-06-30

**Authors:** Marlieke L.M. Jongsma, Ilana Berlin, Ruud H.M. Wijdeven, Lennert Janssen, George M.C. Janssen, Malgorzata A. Garstka, Hans Janssen, Mark Mensink, Peter A. van Veelen, Robbert M. Spaapen, Jacques Neefjes

**Affiliations:** 1 Division of Cell Biology, The Netherlands Cancer Institute, Plesmanlaan 121, 1066 CX Amsterdam, the Netherlands; 2 Department of Immunohematology and Blood Transfusion, Leiden University Medical Centre, P.O. Box 9600, 2300 RC Leiden, the Netherlands; 3 Department of Immunopathology, Sanquin Research and Landsteiner Laboratory AMC/UvA, Plesmanlaan 125, 1066 CX Amsterdam, the Netherlands; 4 Department of Chemical Immunology, Leiden University Medical Centre, P.O. Box 9600, 2300 RC Leiden, the Netherlands

**Keywords:** EGFR signaling, endosomes, EPS15, E3 ligase, ER, lysosomes, perinuclear, RNF26, TAX1BP1, TGN, TOLLIP, transport, ubiquitin, USP15

## Abstract

Through a network of progressively maturing vesicles, the endosomal system connects the cell’s interior with extracellular space. Intriguingly, this network exhibits a bilateral architecture, comprised of a relatively immobile perinuclear vesicle “cloud” and a highly dynamic peripheral contingent. How this spatiotemporal organization is achieved and what function(s) it curates is unclear. Here, we reveal the endoplasmic reticulum (ER)-located ubiquitin ligase Ring finger protein 26 (RNF26) as the global architect of the entire endosomal system, including the *trans*-Golgi network (TGN). To specify perinuclear vesicle coordinates, catalytically competent RNF26 recruits and ubiquitinates the scaffold p62/sequestosome 1 (p62/SQSTM1), in turn attracting ubiquitin-binding domains (UBDs) of various vesicle adaptors. Consequently, RNF26 restrains fast transport of diverse vesicles through a common molecular mechanism operating at the ER membrane, until the deubiquitinating enzyme USP15 opposes RNF26 activity to allow vesicle release into the cell’s periphery. By drawing the endosomal system’s architecture, RNF26 orchestrates endosomal maturation and trafficking of cargoes, including signaling receptors, in space and time.

## Highlights

• The bulk of the endosomal system and TGN clusters in the perinuclear “cloud”
• The ER-located E3 ubiquitin ligase RNF26 retains vesicles in the perinuclear cloud
• RNF26 employs the ubiquitin scaffold SQSTM1 to capture specific vesicle adaptors
• Opposition between RNF26 and DUB USP15 times release of vesicles for fast transport

## Graphical Abstract

**Figure:**
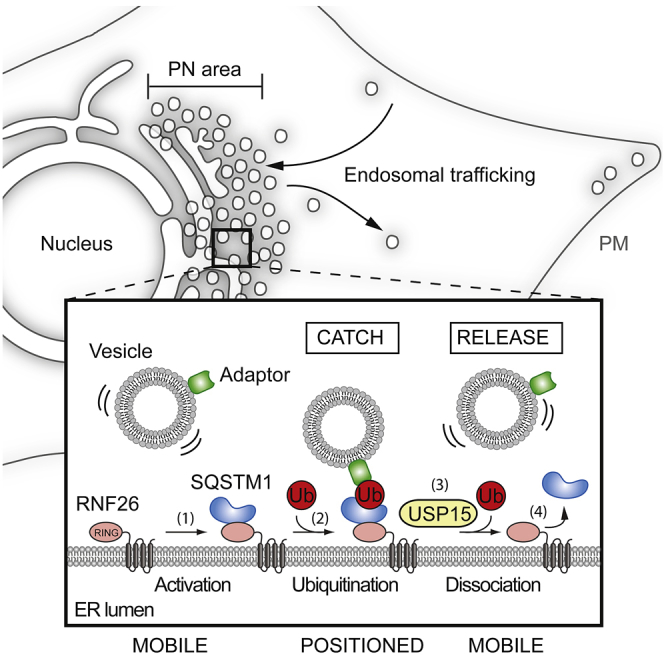

## Teaser

The endosomal system exhibits a bilateral architecture, comprised of a relatively immobile perinuclear vesicle “cloud” and a highly dynamic peripheral contingent. How this cloud is organized and what purpose it serves is unknown. Here, we reveal its molecular determinants centered around the ER-located ubiquitin ligase RNF26, capable of retaining the entire endosomal system’s repertoire through a common mechanism operating at the ER membrane. Countered by the deubiquitinating enzyme USP15, RNF26 draws the endosomal system’s architecture, thus orchestrating vesicle maturation and cargo trafficking in space and time.

## Introduction

Visual inspection of a typical cell reveals canonical arrangements of membrane-enclosed organelles. Generally, the endoplasmic reticulum (ER) wraps around the nucleus, extending throughout the cytoplasmic space, while the mammalian Golgi stacks cluster between the nucleus and the microtubule-organizing center (MTOC) (Rios and Bornens, 2003, Thyberg and Moskalewski, 1999, Valderrama et al., 1998). From here, the *trans*-Golgi network (TGN) vesicles disseminate biosynthetic cargoes to their sites of function throughout the cell (Waguri et al., 2003). On the other side of vesicle traffic, endosomes originating from the plasma membrane carry internalized cargoes to the lysosome for degradation or spare them through diversion to recycling (Raiborg and Stenmark, 2009). The roads traveled by endosomes time intracellular signaling cascades (Scita and Di Fiore, 2010) and tune specialized functions, such as antigen processing and pathogen clearance in immune cells (Blum et al., 2013). To fulfill its myriad responsibilities, the collective endo- and exocytic pathway connects distant organelles (Traub and Bonifacino, 2013) through a progressively maturing network of vesicles (Huotari and Helenius, 2011). How cells sense and manipulate the location of individual vesicles in space and time to suit their global housekeeping and environmental demands is unclear.

Intriguingly, the bulk of lysosomes, early and late endosomes, as well as vesicles of the TGN, locates quiescently in the perinuclear region of the cell (Anikeeva and Sykulev, 2011, Reed et al., 2013, Rojo Pulido et al., 2011, Sadacca et al., 2013, Wasmeier et al., 2008), poised toward the cell’s periphery. Only select vesicles escape this perinuclear (PN) “cloud” to become subject to fast bidirectional transport (Wubbolts et al., 1999) by dynein (Cantalupo et al., 2001, Jordens et al., 2001, Schroeder et al., 2014) and kinesin motors (Raiborg et al., 2015, Rosa-Ferreira and Munro, 2011). What governs acceptance of vesicles into—and their release from—the PN cloud is unknown. Gaining fundamental insights into the way such decisions are made in molecular terms is pivotal to understanding regulation of transport programs in the cell.

The ER is the only intracellular organelle that occupies every corner of cytosolic space. Not surprisingly, it has been shown to participate in various functional contacts with other membranous compartments, mediating exchange of metabolites and controlling transport and fusion processes (Helle et al., 2013). Currently, contact sites between the ER and endosomes are emerging as potent regulatory hubs for vesicle transport (Raiborg et al., 2015, Rocha et al., 2009), fusion (van der Kant et al., 2013), and fission (Rowland et al., 2014) events. Here, we describe how an ER-associated protein network, organized by the E3 ubiquitin ligase Ring finger protein 26 (RNF26), serves as a platform for perinuclear positioning of the entire endosomal system. Localized in the ER membrane, RNF26 extends its catalytic determinants into the cytosol, restricting fast microtubule-based transport of early, recycling, and late endosomes/lysosomes and the TGN. We show that RNF26 utilizes the ubiquitin scaffold p62/sequestosome 1 (SQSTM1) as its substrate to attract ubiquitin-binding domains (UBDs) of various vesicle membrane adaptors. The resulting molecular bridge restrains cognate vesicles in the perinuclear region and organizes the endosomal pathway for efficient cargo transfer and ligand-induced clearance of signaling receptors. Vesicles can then be released for fast transport into the cell’s periphery from their perinuclear positions by way of RNF26-associated deubiquitinating enzyme (DUB) USP15, thus completing the dynamic cycle. Collectively, the RNF26-based protein network elucidates a key paradigm for functional control of intracellular architecture and organelle dynamics, highlighting the importance of inter-compartmental regulation in membrane cell biology.

## Results

### RNF26 Regulates Endocytic Compartment Architecture and Dynamics

**Figure 1:**
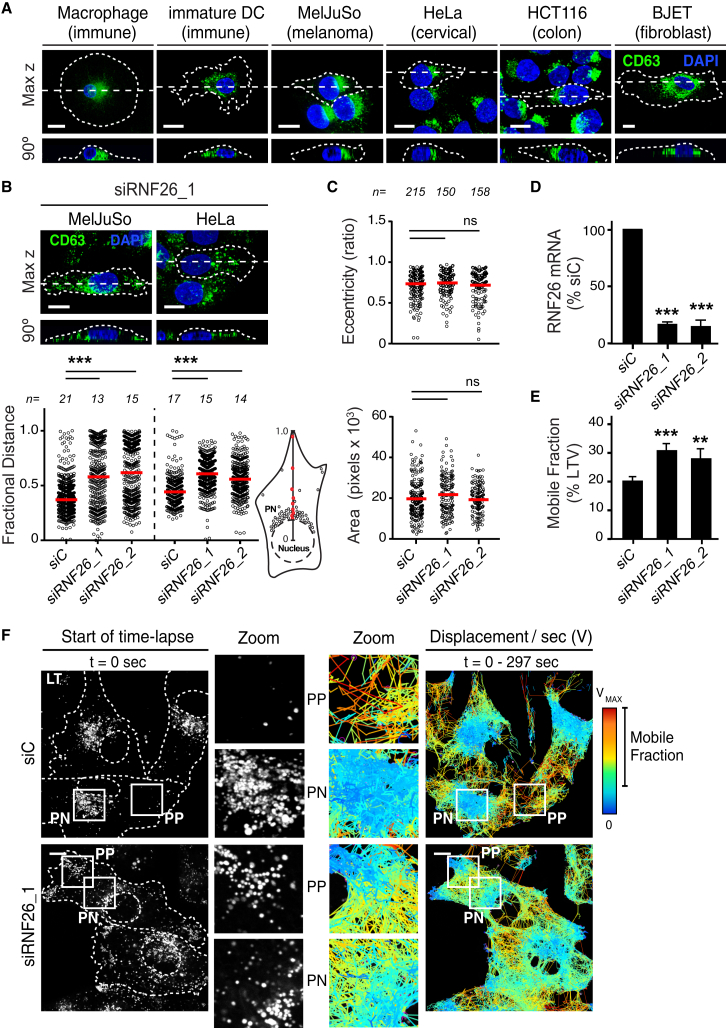
RNF26 Depletion Disrupts Spatiotemporal Organization of Endosomes (A) Intracellular distribution of LEs (CD63, green) in various cell lines. Representative maximum z projection (3D) overlays with nuclear DAPI (blue) and their corresponding z cross sections along the demarcated line are shown. Cell boundaries are depicted in dashed lines. For other markers, see Figure S1. (B) Effect of RNF26 depletion on distribution of LEs, represented as fractional distances of CD63 vesicles from center of nucleus (distance of pixels from nucleus = faction of distance from nucleus to the plasma membrane [1.0]; mean shown in red). For 3D view, see Movies S1A and S1B. (C) Cell shape analysis for samples in (B), showing total cell area and eccentricity calculated in an automated fashion as described in the Supplemental Experimental Procedures. (D) mRNA levels of RNF26 targeted by two different siRNAs (siRNF26_1 and siRNF26_2) as assessed by qPCR are expressed relative to siC; n = 3. (E) Quantification of the mobile fraction of acidified Lysotracker (LT)-positive vesicles (LTVs) as a function of RNF26; n = 3. For details, refer to the Supplemental Experimental Procedures. (F) Organization and dynamics of LTVs (white) in control (siC) versus RNF26-depleted (siRNF26_1) MelJuSo cells. Left panels: representative single confocal plane fluorescence images taken at the start of time lapse. Right panels: vesicle displacement rates (blue, immobile; red, max mobility) observed over the 297-s time interval. Nuclei and cell boundaries are depicted in dashed lines, and zoom-ins highlight peripheral (PP) and perinuclear (PN) boxed regions. Quantification appears in (E). For LT time lapses, see Movies S2A–S2C. For CD63 time lapses see Movies S2D and S2E. Scale bars, 10 μm. For all figures: *n*, # of cells analyzed per condition; n, # independent experiments; error bars, SD.

**Figure S1:**
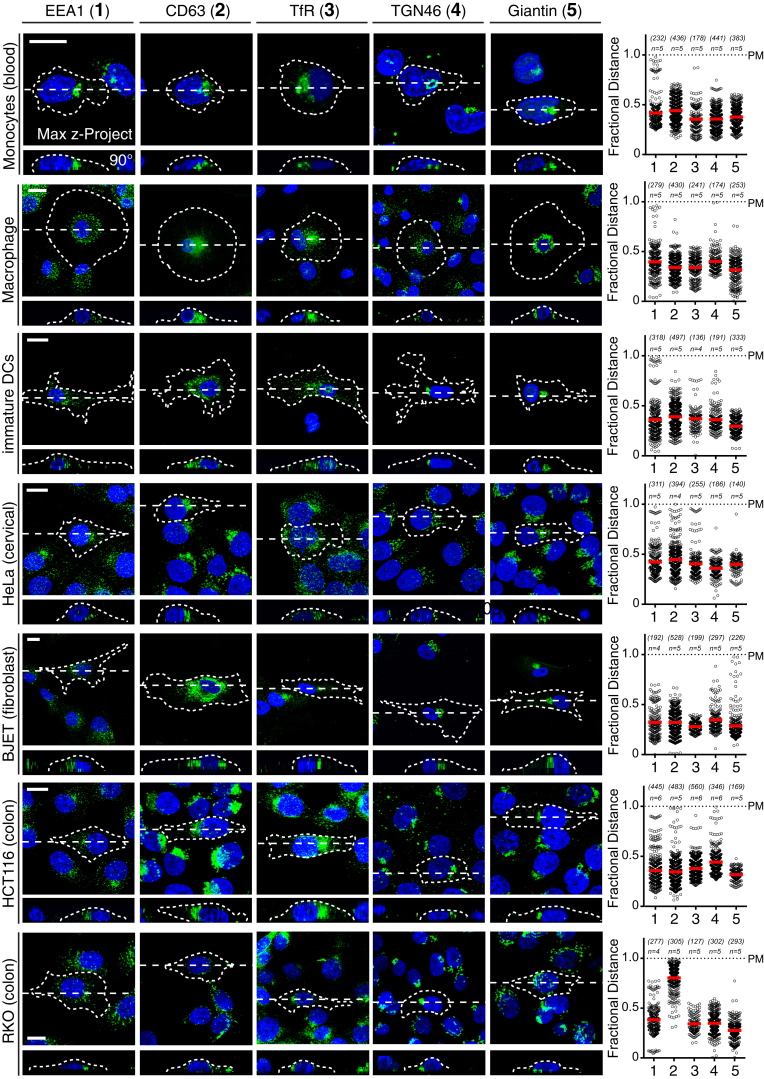
A Wallpaper Showing Endosomal and Golgi Distribution in Various Cell Types, Related to Figure 1 Intracellular distribution of early endosomes (EEA1 (1), green), LEs/Lysosomes (CD63 (2), green), recycling endosomes (TfR (3), green), Trans-Golgi network (TGN46 (4), green), and Golgi (Giantin (5), green) in various cell lines (3 human primary immune cells, monocytes, macrophages, and immature dendritic cells, and 4 human cell lines including the primary BJET fibroblast cell line). Representative maximum z-projection (3D) overlays with nuclear DAPI (blue) and their corresponding z-cross sections along the demarcated line are shown below the X-Y images. Cell boundaries are depicted in dashed lines. Fractional distance analysis of marked vesicles in various cell lines are shown (right panels) reported as distance of pixels from nucleus = faction of distance from nucleus to the plasma membrane (max 1.0); mean shown in red. Numbers below the figure relate to the marker proteins indicated above the microscopy images; n = 2; scale bar, 10 μm; *n* = # of cells analyzed per condition, with # of data points analyzed per condition given in parenthesis; n, # independent experiments.

Across cell types, a wide variety of endosomal maturation stages—late (CD63, Figure 1A), early (EEA1) and recycling (TrfR) endosomes, as well as the vesicular arm of the TGN (TGN46) (Figure S1)—tend to cluster into a “cloud” near the nucleus, with only a fraction of each subset extending into the cell’s periphery. How such organization is established and controlled and what purpose it may serve is largely unknown. Given that late endosomes (LEs) constitute central nodes within the endo- and exocytic vesicular network (Huotari and Helenius, 2011), we mined a genome-wide small interfering RNA (siRNA)-based screen for novel factors controlling LE biology (Paul et al., 2011), where silencing the RING finger ubiquitin ligase RNF26 was shown to severely disrupt the intracellular LE organization, leading to marked dispersion of LEs throughout the cytoplasm and even to the tips of cells, without significantly impacting cell shape (Figures 1B–1D; Movies S1A and S1B). These observations cast RNF26 as a potent candidate for control of the LE compartment architecture, prompting us to investigate the role of RNF26 in the organization and function of the perinuclear (PN) cloud.

In live cells, we observed a striking relationship between the LE compartment architecture and its dynamics. The majority of acidified vesicles marked by Lysotracker (LTVs) were positionally restricted to the PN cloud, while the sparsely populated periphery (PP) remained dynamic over time (Figure 1F, top right panels; Movie S2A). On the contrary, cells depleted of RNF26 exhibited an expanded periphery and increased mobility of the LTV contingent relative to control, thus blurring the PN/PP distinction (Figures 1E and 1F, bottom right panels; Movies S2B and S2C, Lysotracker; Movies S2D and S2E, mCherry-CD63).

**Figure 2:**
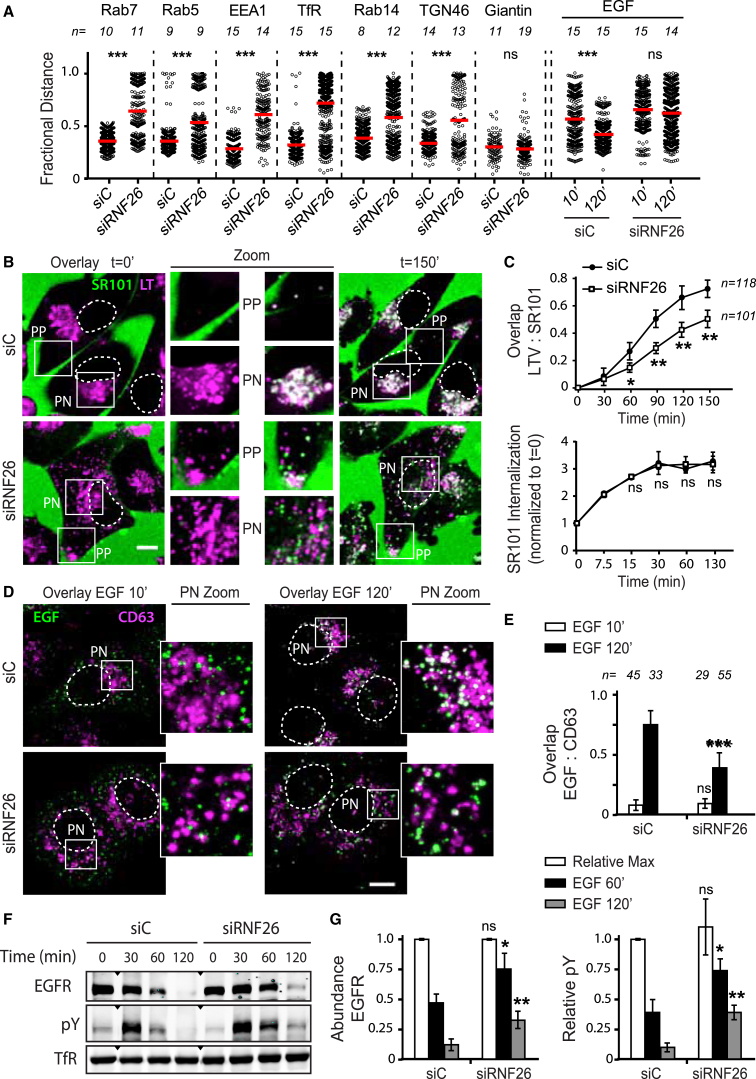
RNF26 Promotes Cargo Trafficking and Endosome Maturation in the Perinuclear Cloud (A) Intracellular distribution of various markers and cargoes in MelJuSo cells in response to RNF26 depletion, presented as fractional distances with mean shown in red, as in Figure 1B. Late endosomes, Rab7; early endosomes, EEA1, Rab5, and Rab14; recycling endosomes, Transferrin receptor (TfR); TGN, TGN46; Golgi, Giantin; ligand-mediated endocytosis, EGF. See also Figures S2A, S2B, and 2D and Movies S3A–S3D. (B) Trafficking of fluid phase dye SR101 (green) to the acidified compartment (LT, magenta). Single confocal plane fluorescence overlays with PP and PN zooms at t = 0 and t = 150 min following addition of SR101 to control or RNF26-depleted MelJuSo cells are shown. See also Movies S4A and S4B. (C) Top graph: quantification (Mander’s overlap) of SR101 entry into LTVs as a function of time (min) in control MelJuSo cells or those silenced for RNF26. n = 2. Bottom graph: total uptake of SR101 in control or RNF26-depleted MelJuSo cells as measured by flow cytometry, expressed as fold increase normalized to t = 0 as a function of time; n = 3. (D) Effect of RNF26 depletion on receptor-mediated trafficking of EGF-555 (green) to the late endosome compartment (CD63, magenta). Representative z projection (3D) overlays at 10 min (left panels) and 120 min (right panels) following stimulation (100 ng/ml) are shown with PN zooms for control and RNF26-depleted HeLa cells. (E) Colocalization (Mander’s overlap) of EGF with CD63 at 10 min (white) and 120 min (black) following ligand stimulation; n = 2. (F) EGFR degradation in control versus RNF26-depleted HeLa cells. Immunoblots against total (EGFR) and phosphorylated (pY) EGFR, as well as TfR (loading control) along a time course following 20 ng/ml EGF addition (min) are shown (lane corresponding to t = 10 min was excised from siC panel). For analysis of surface and total EGFR levels see Figures S2C and S2E, respectively. (G) Quantification of total (left graph, relative to t = 0) and activated (right graph, pY relative to siC at t = 30′) EGFR as a function of time following EGF addition; n = 3. Scale bars, 10 μm.

**Figure S2:**
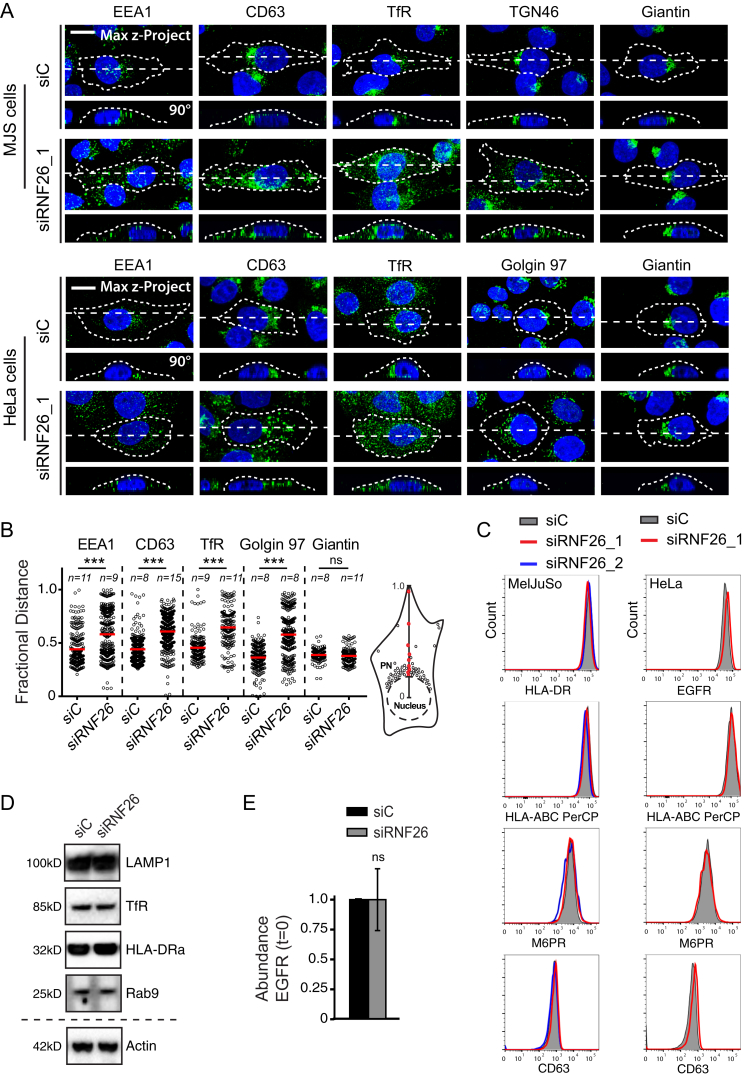
Effect of RNF26 Depletion on the Expression and Distribution of Various Marker Proteins, Related to Figure 2 (A) MelJuSo cells (top panels) and HeLa cells (bottom, panels) transfected with siRNA against RNF26 (siRNF26_1) or control siRNA (siC), as indicated. Intracellular distribution of Early endosomes (EEA1, green), LEs/Lysosomes (CD63, green), recycling endosomes (TfR, green), Trans-Golgi network (TGN46, green) and the Golgi (Giantin, green) are shown. Representative maximum z-projection (3D) overlays with nuclear DAPI (blue) and their corresponding z-cross sections along the demarcated line are shown below the X-Y images. Cell boundaries are depicted in dashed lines; n = 2. (B) Intracellular distribution of various markers and cargoes in HeLa cells in response to RNF26 depletion (siRNF26), shown in (A), presented as fractional distances with mean shown in red. For MelJuSo cells, see Figure 2A. (C) Cell surface expression of various markers in RNF26-depleted MelJuSo cells (left panels, MHC class II (HLA-DR), MHC class I (HLA-ABC), M6PR and CD63) or HeLa cells (right panels, EGFR, MHC class I (HLA-ABC), M6PR and CD63) analyzed by flow cytometry; n = 3. (D) Total levels of endosomal marker proteins and cargoes in MelJuSo cells depleted of RNF26 (siRNF26) compared to control cells (siC) analyzed by SDS-PAGE and WB using β-actin as loading control. Lysosomes (LAMP1); recycling endosomes (TfR); MIIC (MHC class II: HLA-DRa) and (late)-endosomes (Rab9). (E) Total cellular abundance of EGFR in HeLa cells depleted of RNF26 (siRNF26) compared to control cells (siC) analyzed by SDS-PAGE and WB using TfR as loading control; n = 3. Scale bar, 10 μm; *n* = # of cells analyzed per condition, n = # independent experiments, error bars = SD.

To test whether other components of the endo- and exocytic vesicular repertoire also fall under the RNF26 purview, we investigated the effect of RNF26 silencing on distribution of various vesicle markers. Without exception, localization of all post-Golgi vesicles examined was susceptible to RNF26 depletion in two different cell lines tested, while distribution of the Golgi remained unaffected (Figures 2A, S2A, and S2B; Movies S3A–S3D). Given that intracellular organization and its associated compartmentalization of transport apply across diverse vesicle types, we hypothesized that the PN cloud may serve as a meeting hub for maturation and cargo exchange. In support of this, we found that vesicles endocytosed by fluid-phase, as monitored using uptake of the extracellular dye sulforhodamine (SR101) (Wubbolts et al., 1996), readily encountered LT-positive structures residing primarily in the PN cloud. By contrast, acquisition of SR101 by the disorganized acidified compartments in RNF26-depleted cells was markedly restrained (Figures 2B and 2C; Movies S4A and S4B), while SR101 internalization rate remained unaffected (Figure 2C), suggesting that endosomes mature in the PN cloud. We further explored whether trafficking of specific cargoes to the proteolytic compartment is affected by the endosomal system’s architecture. Following acute stimulation with EGF, ligand-containing vesicles distributed to the PN cloud over time in control cells, but not in those compromised for RNF26 (Figure 2A). In the latter case, trafficking of EGF-positive vesicles to the LE compartment was severely impaired (Figures 2D and 2E), while availability of EGF receptor (EGFR) on the cell surface, as well as total receptor levels, remained unaffected (Figures S2C and S2E). Consistent with the above, ligand-induced degradation of EGFR was attenuated with RNF26 depletion, leaving activated receptors (detected as pY) to linger at late time points following stimulation (Figures 2F and 2G). Taken together with the SR101 experiments, these findings imply that the PN cloud and its architect RNF26 facilitate efficient vesicle maturation and transit of cargo through the endosomal system, with implications for ligand-induced receptor signaling.

### ER Localization and Ubiquitin Ligase Activity of RNF26 Mediate Endosomal Positioning

**Figure 3:**
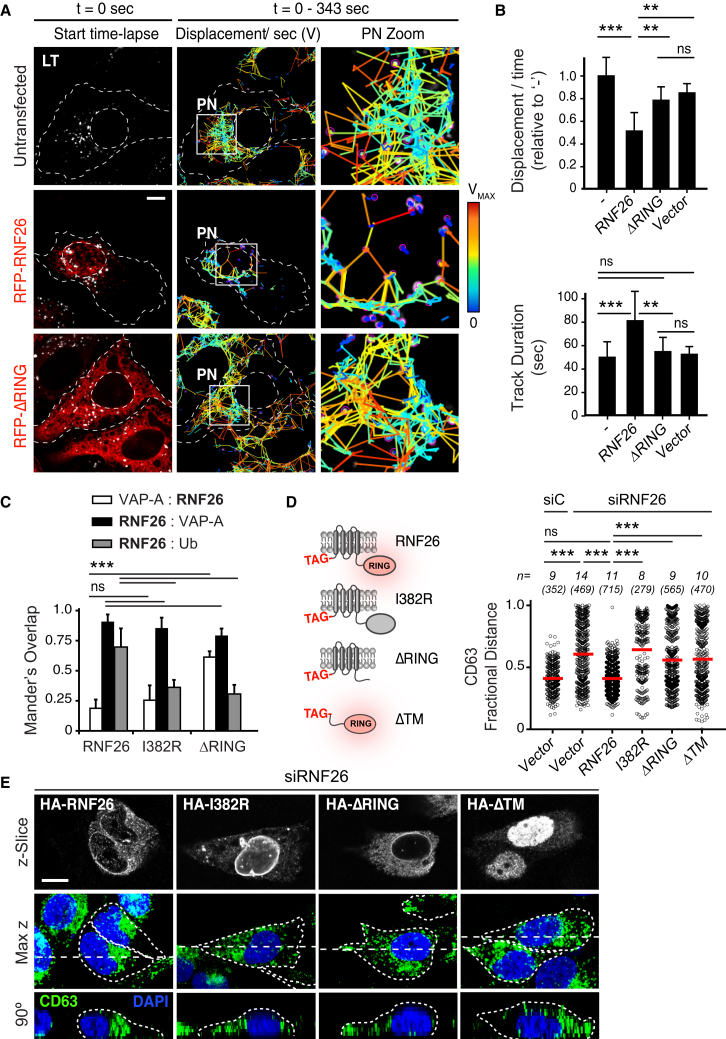
ER-Associated Ubiquitin Ligase Activity of RNF26 Organizes the PN Cloud and Controls Vesicle Dynamics (A) Effect of RNF26 on vesicle dynamics. Left panels: representative single confocal plane fluorescence overlays of LT (white) in control (untransfected; focal plane through the nucleus) HeLa cells or those ectopically expressing (red) RFP-RNF26 (focal plane just above the nucleus) or its ΔRING mutant (focal plane through the nucleus) at the start of time lapse are shown. Middle panels: corresponding vesicle displacement rates (blue, immobile; red, max mobility) observed during the 343-s time interval. Right panels: zooms of boxed PN regions. (B) Quantification of displacement rates (relative to untransfected cells) and track duration times (s) for data presented in (A); n = 4. (C) Colocalization (Mander’s overlap) of RNF26 or its mutants I382R and ΔRING with the ER protein VAP-A or ubiquitin. White bars, overlap VAP-A with RNF26; black bars, overlap RNF26 with VAP-A; gray bars, overlap RNF26 with ubiquitin; *n* = 10 cells per sample per experiment, n = 3. See also Figures S3A–S3C. (D) Rescue of RNF26 depletion phenotype (siRNF26 targeting 3′UTR) by re-expression of wild-type HA-RNF26, its RING domain mutants I382R and ΔRING, its *trans*-membrane truncation ΔTM or empty vector. Late endosome (LE) fractional distance analysis of CD63 in MelJuSo cells is shown (mean in red; number of data points analyzed per condition in parenthesis), along with a schematic overview of RNF26 constructs used. For cell shape analysis of ectopic RNF26 expression, see Figure S3D. (E) Selected representative maximum z projection (3D) image overlays of CD63 (green) with nuclear DAPI (blue) corresponding to quantification in (D) are shown, together with their z cross sections along the demarcated line. Single confocal slices in top panels show HA-tagged expression. Scale bars, 10 μm.

**Figure S3:**
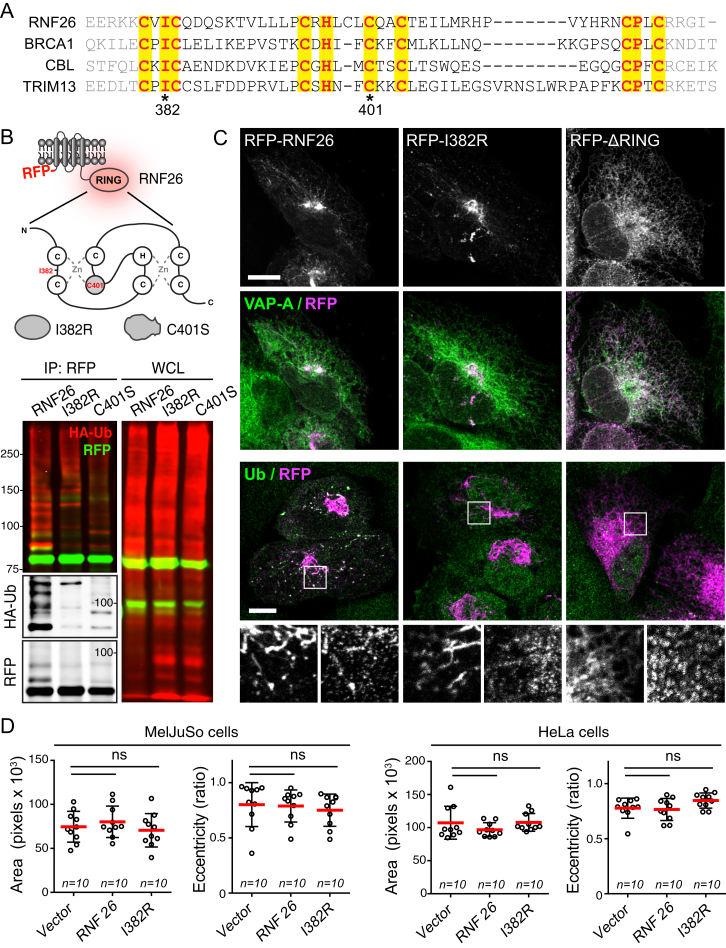
The RING Domain of RNF26 Mediates Its Localization and Function, Related to Figure 3 (A) Sequence comparison of the RNF26 RING domain with the RING domains of three other structurally related E3-ligases (BRCA1, CBL and TRIM13). The amino acids required for the Zn^2+^ positioning are indicated in yellow; amino acids mutated to inactivate RNF26 are indicated with ^∗^ (Iso 382 and Cys 401). (B) (top) Schematic overview of the RNF26 RING domain: residues Iso382 and Cys401 mutated in this study are indicated in red. (bottom) RFP-RNF26 and its inactive mutants, co-expressed with HA-Ubiquitin in HEK293 cells, were immunoprecipitated under harsh lysis conditions, separated by SDS-PAGE and analyzed by WB to visualize the associated modifications with HA-Ub (left panel). Whole-cell lysate (WCL) controls are shown on the right. (C) RFP-RNF26 or the indicated mutants (magenta) were expressed in MelJuSo cells and co-stained for VAP-A (green) to label the ER or with Ubiquitin (green). Zoom-in of the indicated region illustrates effective co-labeling of WT, but not catalytically incompetent RNF26 with Ub. (D) Effect of ectopical expression of RNF26 or its inactive mutant I382R on the size and eccentricity in either MelJuSo (left panels) or HeLa cells (right panels) (mean shown in red); n = 2. *n* = # of cells analyzed per condition, n = # independent experiments, error bars = SD; scale bar, 10 μm.

Having determined that loss of RNF26 incurs detrimental effects on endosomal organization and function, we turned to ask whether this ER-located ubiquitin ligase (Qin et al., 2014) actively positions endosomes in the PN cloud. Ectopic expression of full-length RNF26, but not its catalytic RING domain (ΔRING) truncation, substantially restricted mobility of LT-positive vesicles (Figures 3A and 3B). Mirroring the PN position of the vesicle cloud, RNF26 localized predominantly to the region of the ER proximal to the nucleus, while its ΔRING mutant distributed throughout the ER (Figures 3C and S3A–S3C; ER marked by VAP-A), indicating that the RING domain drives retention of the ligase in the perinuclear ER subdomain.

To explore the contribution of catalytic activity to RNF26 localization and function, we mutated a conserved Isoleucine 382 to Arginine (I382R, Figure S3A), thereby inhibiting expected interactions with E2 enzyme(s) without incurring deleterious effects on RING domain architecture, such as by mutating key Zn^+2^ coordinating modules (Deshaies and Joazeiro, 2009). RNF26-I382R markedly reduced the enzyme’s ubiquitin ligation capacity (Figure S3B), similar to the previously reported C401S mutant (Qin et al., 2014). Ubiquitin ligase activity was further illustrated by strong colocalization of wild-type RNF26 with ubiquitinated species, relative to its catalytically dead mutants showing only marginal overlap with ubiquitin (Figures 3C and S3C). We next tested whether ubiquitin ligase activity afforded by RNF26 is critical to perinuclear endosome positioning. The RNF26 depletion phenotype, scored on the basis of LE scattering away from the nucleus, was robustly rescued by re-expression of RNF26, but not its mutants deficient in either ubiquitination or ER transmembrane segments (Figures 3D and 3E), implying that RNF26-mediated ubiquitination must take place at the ER membrane to effectively position vesicles in the PN cloud.

### RNF26 Interacts with a Network of Vesicle-Associated Adaptor Proteins

**Figure 4:**
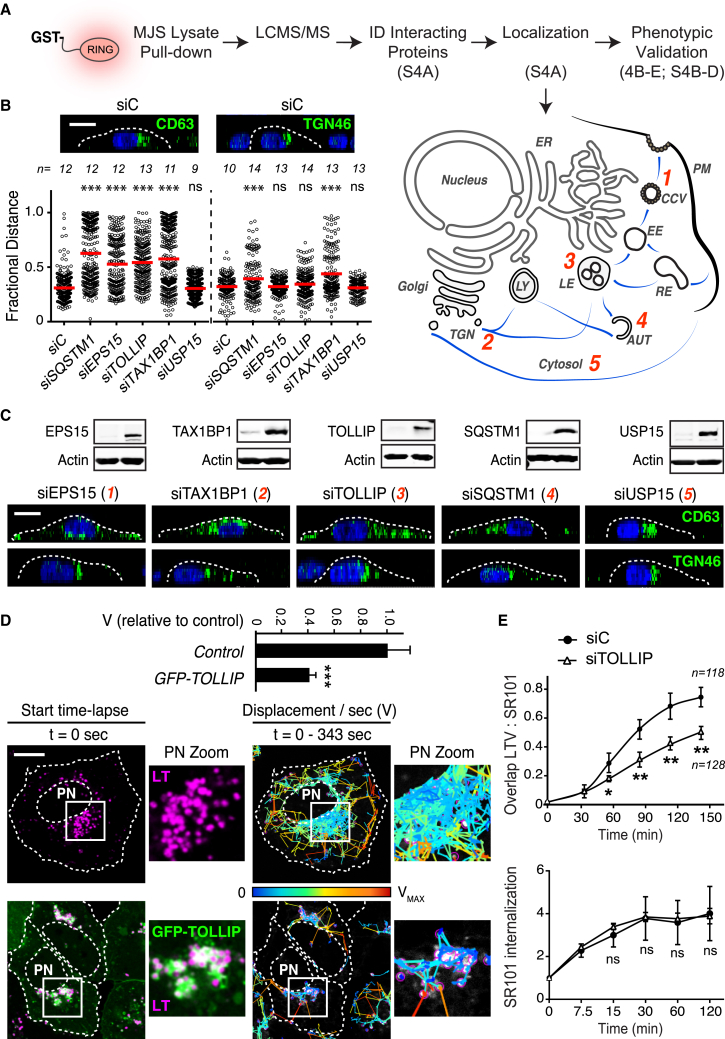
Protein Network Associated with the RING Domain of RNF26 (A) Workflow scheme for the identification and validation of proteins interacting with the cytosolic domain of RNF26. RING-associated RNF26 proteome consists of membrane-associated adaptor proteins and a DUB USP15 (for proteomic analysis details, see Figure S4A). Localization of RNF26-interacting proteins EPS15, TOLLIP, TAX1BP1, SQSTM1, and USP15 are depicted schematically (for representative marker overlays see Figure S4A). CCV, clathrin-coated vesicle; EE, early endosome; RE, recycling endosome; LE, late endosome; Ly, lysosome; TGN, *trans*-Golgi network; AUT, autophagosome; PM, plasma membrane. (B) Intracellular distribution (fractional distance analysis, mean shown in red) of LEs (CD63) and TGN (TGN46) in MelJuSo cells as a function of indicated siRNA perturbations. For cell shape analysis, see Figure S4B. For combinatorial silencing of vesicle adaptors, see Figure S4C. (C) Representative z-cross section (3D) image overlays of CD63 (green, upper panels) or TGN46 (green, bottom panels) with nuclear DAPI (blue) in MelJuSo cells are shown with the corresponding protein levels of silenced targets (left lanes) as compared to the control (right lanes). (D) Effect of GFP-TOLLIP (green) overexpression on the organization and dynamics of acidified vesicles (LTVs, magenta) in HeLa cells. Left panels: representative single confocal plane fluorescence image overlays taken at the start of the time lapse. Right panels: corresponding vesicle displacement rates (blue, immobile; red, max mobility) observed during the 343-s time interval; zoom-ins highlight boxed PN regions. Quantification of LTV dynamics (displacement/s relative to untransfected cells) as a function of TOLLIP is shown above the images; n = 2. See also Figure S4D and Movies S5A and S5B. (E) Top graph: quantification (Mander’s overlap) of SR101 entry into acidified vesicles (LTVs) as a function of time (min) in control MelJuSo cells (siC; control dataset in common with Figure 2C) versus those depleted of TOLLIP (siTOLLIP). n = 2. Bottom graph: total uptake of SR101 in control or TOLLIP-depleted MelJuSo cells as measured by flow cytometry, expressed as fold increase normalized to t = 0 as a function of time; n = 3. Scale bars, 10 μm.

**Figure S4:**
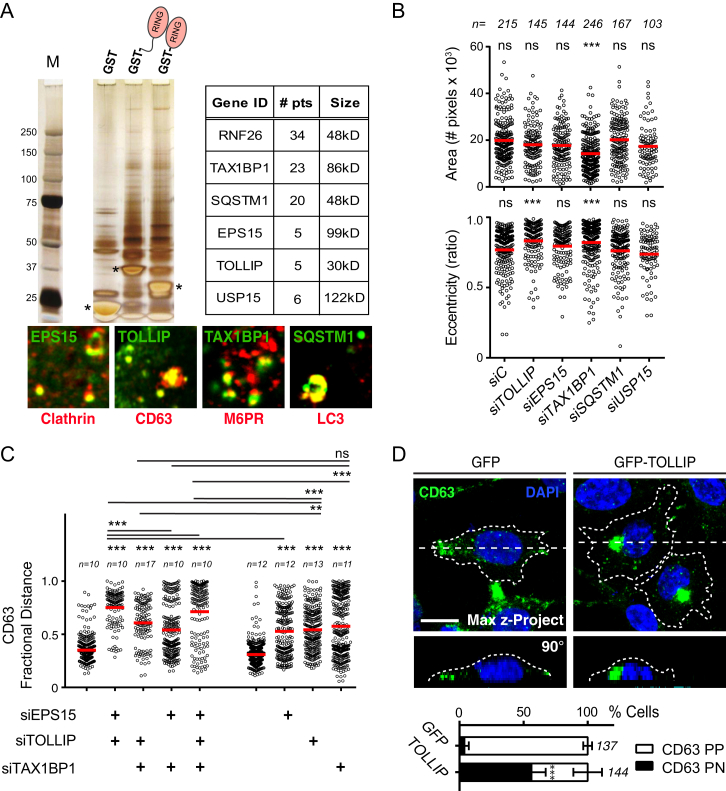
Isolation and Characterization of Proteins Associated to the RNF26 Cytoplasmic Domain, Related to Figure 4 (A) (left) Isolation and characterization of RNF26 associated proteome interacting with its RING domain. Two GST-tagged RNF26 tails (aa304-433 and aa363-433) including the RING domain were used (last two lanes) and compared to GST-tag only as a control (first lane). The Silver-stained gel (including marker lane) with bands isolated and identified by mass spectrometry marked as indicated. (right) Table showing the number of unique identified peptides (#pts) corresponding to RNF26 and its interacting partners TAX1BP1, SQSTM1, EPS15, TOLLIP and USP15 that were absent in the GST-only control lane. (bottom) Zoom-in of identified endogenous proteins along with marker proteins for their vesicular localization in MelJuSo cells. EPS15 (green) marks clathrin-coated vesicles (clathrin, red); TOLLIP (green) locates to late endosomes (CD63, red); TAX1BP1 partially co-localizes with secreted M6PR (red) and SQSTM1 locates to autophagosomes (LC3, red). (B) Effect of protein depletion (identified in (A) on cell size and shape (eccentricity) in MelJuSo cells compared to control cells (siC) with mean shown in red; n = 2. (C) Fractional distance analysis of CD63 marked late endosomes in MelJuSo cells after silencing one or multiple ubiquitin adaptors identified. Mean in red and statistical significance relative to the control (siC) are indicated. (D) In contrast to other cell types analyzed, RKO tumor cell line displayed unusual distribution of CD63 (green, upper panel, see also Figure S1). Overexpression of GFP-TOLLIP in these cells re-clusters CD63 positive vesicles (green, bottom panel). Representative maximum z-projection (3D) overlays with nuclear DAPI (blue) and their corresponding z-cross sections along the demarcated line are shown below the X-Y images. Cell boundaries are depicted in dashed lines. GFP signal was omitted from the presented image, but use to define the transfected cells. Quantification of perinuclear (PN) and peripheral (PP) positioning of CD63 positive vesicles following GFP or GFP-TOLLIP expression; n = 2. Scale bar, 10 μm; *n* = # of cells analyzed per condition, n = # independent experiments, error bars = SD.

To understand how an ER-located protein exerts control over the endosomal system and the TGN, we sought out interacting partners of the cytosolic tail of RNF26. Mass spectrometric analysis of proteins co-precipitating with either GST-ΔTM or GST-RING (Figures 4A and S4A) identified three membrane-associated adaptor proteins functioning in sorting and trafficking of endo- or exocytic vesicles—EPS15 (Benmerah et al., 1999), T6BP/TAX1BP1 (Morriswood et al., 2007), and TOLLIP (Ankem et al., 2011), a ubiquitin scaffold p62/SQSTM1 (Ciani et al., 2003) known for its role in autophagy (Lippai and Löw, 2014) and a DUB USP15, which localizes to the nucleus and cytosol, targeting the transforming growth factor β (TGF-β) and nuclear factor kappa-light-chain enhancer of activated B cells (NF-κB) pathways (Eichhorn et al., 2012, Schweitzer et al., 2007). Collectively, cargo specificities of the three former proteins afford broad coverage of both endocytic and biosynthetic vesicle trajectories (Figures 4A and S4A), implying that by association with different vesicle-targeting adaptors, RNF26 may influence positioning of a wide range of endosomes and the TGN. Silencing the above proteins (excluding USP15) produced marked LE dispersion (Figures 4B and 4C). By contrast, TGN vesicle dispersion resulted only from depletion of the TGN-associated adaptor TAX1BP1 and SQSTM1, but not of endocytic adaptors EPS15 and TOLLIP (Figures 4B and 4C), and overall cell shape parameters were profoundly altered only by depletion of TAX1BP1 (Figure S4B). Further, co-silencing multiple adaptors resulted in additive effects on CD63 distribution (Figure S4C), underscoring the contribution of multi-directional traffic to the global architecture of the LE compartment.

To assess whether specific adaptors can influence localization and dynamics of their cognate vesicles, we followed the mobility of LTVs in cells ectopically expressing GFP-TOLLIP. Double-positive vesicles were found to localize primarily in the PN cloud (Figure 4D), and overall LTV movement was dramatically restricted relative to control (Figure 4D; Movies S5A and S5B). Additionally, exogenous TOLLIP restored PN localization of vesicles marked by CD63 in an exceptional cell line (RKO, Figure S4D), which exhibits natural dispersion of the LE compartment (Figure S1). Mirroring the dysfunction in endosomal maturation incurred by depletion of RNF26 (Figures 2B and 2C), silencing TOLLIP inhibited access of SR101-containing endosomes to the acidified compartments, without affecting the internalization rate (Figure 4E). Taken together, the above observations illustrate the capacity of a specific adaptor to position its chosen vesicles in the PN cloud.

### Catalytically Active RNF26 Attracts Ubiquitin-Binding Domains of Endocytic Adaptors

**Figure 5:**
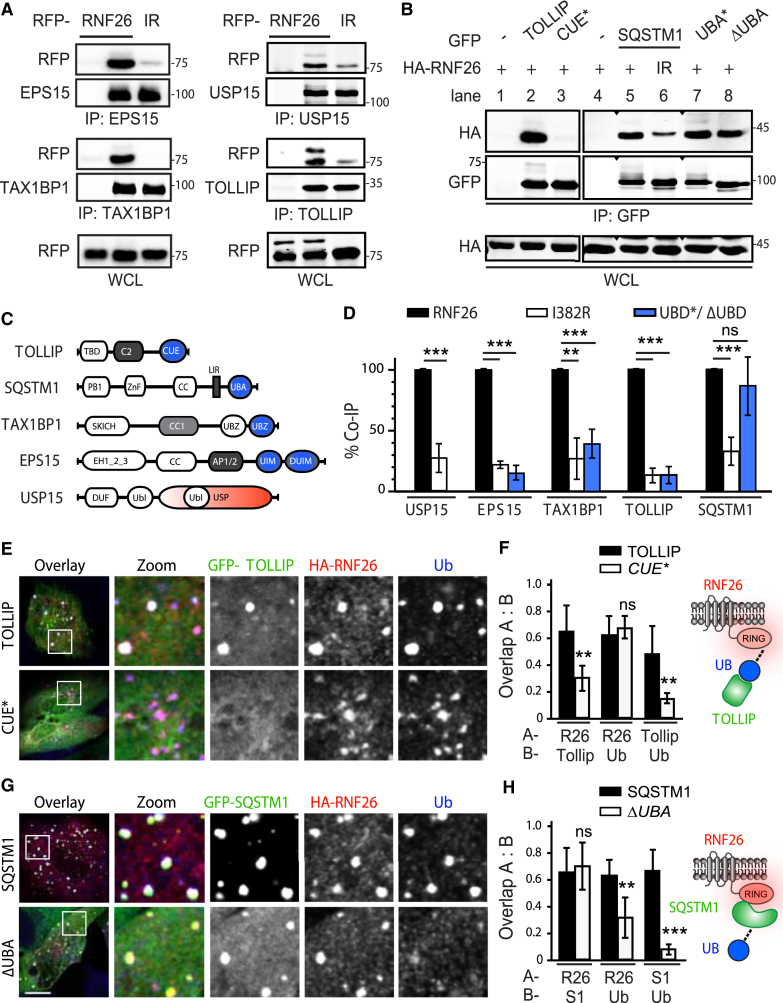
RNF26 Couples to Ubiquitin-Binding Domains of Specific Membrane Adaptors (A) Interactions (assayed by coIP) between RNF26 versus its inactive mutant I382R (IR) and endogenous EPS15, TAX1BP1, TOLLIP, and USP15 in HEK293T cells. WCL, whole-cell lysate. (B) Effects of mutations in ubiquitin-binding domains (UBDs) of TOLLIP and SQSTM1 on interaction with RNF26 in HEK293T cells (extraneous lanes between 4 and 5, as well as 6 and 7 were excised). For TAX1BP1 and EPS15, see Figure S5A. (C) Schematic: domain organization of RNF26-interacting proteins, highlighting membrane-targeting domains (gray), UBDs (blue), and USP (red). (D) Quantification of interactions (normalized as % of WT/WT coIP, black bars) as a function of RNF26 inactivation (I382R white bars) or loss of UBD capabilities (UBA^∗^/ΔUBA, blue bars) for each pair of proteins; n = 3. (E–H) Colocalization of GFP-tagged (green) (E) TOLLIP (quantified in F; n = 2) and (G) SQSTM1 (quantified in H; n = 2) or their UBD mutants (CUE^∗^ and ΔUBA, respectively) with HA-RNF26 (red) and endogenous ubiquitin (blue) in MelJuSo cells. Representative single confocal plane fluorescence overlays and single-channel zooms are shown. Summary is illustrated schematically at the right. Scale bars, 10 μm.

To dissect the molecular basis of communication between RNF26 and its partners, we interrogated their respective interaction determinants. Without exception, EPS15, TAX1BP1, TOLLIP, USP15 (Figure 5A), and SQSTM1 (Figure 5B, right panels) exhibited a strong binding preference for catalytically competent RNF26, relative to the inactive I382R mutant. Given the critical role of RNF26 ubiquitination activity in the establishment of endosomal system’s architecture described in Figure 3, we proceeded to investigate ubiquitin-mediated recognition in this context. Most interacting partners of RNF26 described here harbor ubiquitin-binding domains (UBDs) (Figure 5C), and point mutations targeting the CUE domain of TOLLIP (Mitra et al., 2013), UIM domain of EPS15 (Klapisz et al., 2002), and UBZ2 domain of TAX1BP1 (but not UBZ1, which is incapable of ubiquitin interactions) (Iha et al., 2008) strongly affected association of these proteins with wild-type RNF26 (Figures 5B, left panels, and S5A). Moreover, the UBD-dependent loss of binding was comparable to that observed between wild-type adaptors and mutant RNF26 (Figure 5D). Strikingly, SQSTM1 did not follow suit, displaying no significant reliance on its UBA domain for productive interaction with RNF26 (Figures 5B, right panels, and 5D). Mechanistically, these observations set SQSTM1 apart from the other adaptors within the RNF26 network.

Because the known specificity of SQSTM1 for autophagic membranes is unlikely to account for its broad effects on positioning of both endocytic and biosynthetic systems of vesicles (Figures 4A–4C and S4A), we hypothesized that it may instead function by attracting ubiquitin-dependent partners to RNF26. To test this, we investigated spatial localization of SQSTM1 versus the RNF26-interacting vesicle adaptors relative to the ligase and its associated ubiquitin signals. As expected based on the coimmunoprecipitation (coIP) data, the LE adaptor GFP-TOLLIP readily colocalized with RNF26 at the corresponding sites of endogenous ubiquitin accumulation, while its ubiquitin-binding-deficient point mutant (CUE^∗^) did not (Figures 5E). Importantly, lack of its recruitment to the ligase had no discernable effect on ubiquitin enrichment at RNF26 (Figures 5E and 5F). By contrast, UBA domain truncation of SQSTM1 (ΔUBA) was still able to occupy RNF26-positive structures, but significantly suppressed accumulation of associated ubiquitinated species (Figures 5G and 5H), thus implicating SQSTM1, along with its ubiquitin interactions, in the assembly of ubiquitinated species at RNF26.

### RNF26 and the DUB USP15 Share a Substrate in SQSTM1

**Figure 6:**
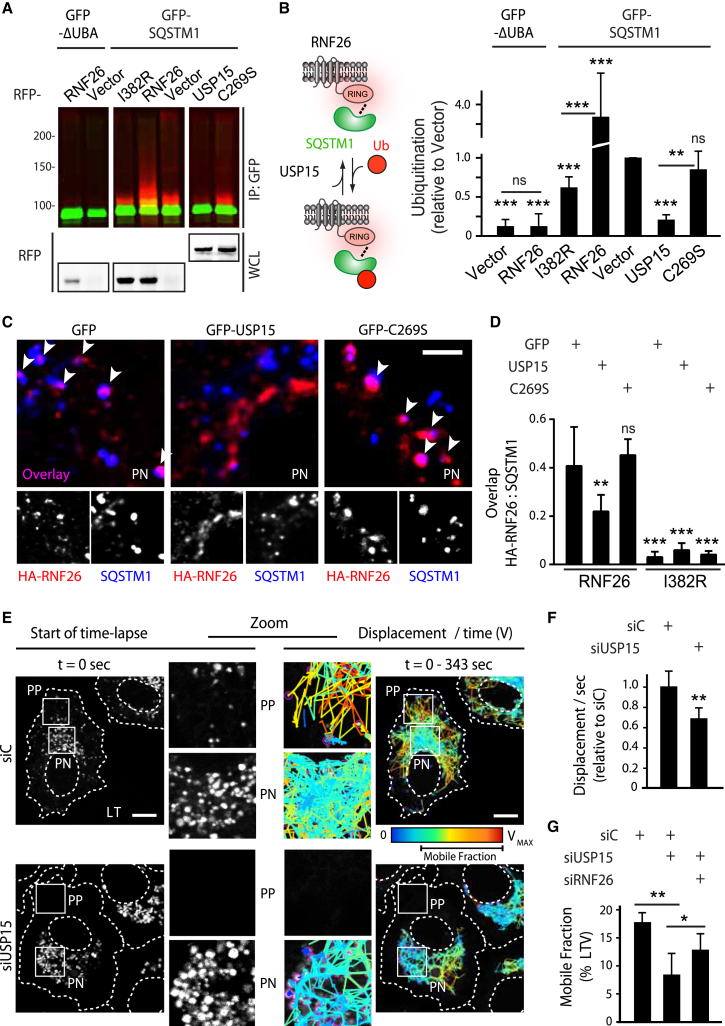
SQSTM1 Is a Substrate for RNF26 and the DUB USP15 (A) Ubiquitination status of SQSTM1 as a function of catalytic activities of RNF26 and USP15. GFP-SQSTM1 (or GFP-ΔUBA) was isolated from HEK293T cells overexpressing HA-Ub in the presence of vector, RFP-RNF26 versus its catalytic mutant I382R, or USP15 versus its catalytic mutant C269S. Ubiquitination status of GFP-substrate (green) was assessed by immunoblots against HA (red). (B) Quantification of the ubiquitination assay in (A); n = 4. Schematic on the left depicts proposed catalytic opposition between RNF26 and USP15. (C) Effect of USP15 on localization of SQSTM1 (blue) at RNF26-positive sites (red) in the PN area. Representative single confocal plane fluorescence overlays of PN regions are shown with their corresponding single channel images (HeLa cells). For a full image panel, see Figure S5B. Scale bar, 2.5 μm. (D) Quantification of RNF26 or I382R occupancy by SQSTM1 (Mander’s overlap) as a function of USP15 catalytic activity; n = 2. (E) Organization and dynamics of acidified LT-positive vesicles (LTVs, white) in control (siC) versus USP15-depleted (siUSP15) HeLa cells. Left panels: representative single confocal plane fluorescence images at the start of time lapse are shown. Right panels: corresponding vesicle displacement rates (blue, immobile; red, max mobility) observed during the 343-s time interval; zoom-ins highlight boxed PP and PN regions. For time lapses, see Movies S5A and S5C. Scale bar, 10 μm. (F) Effect of USP15 depletion on LTV dynamics (displacement/s relative to cells transfected with control siRNA); n = 2. (G) Functional interplay between siUSP15 and siRNF26. Quantification of mobile LTV fraction as a function of indicated siRNA perturbations (+) in MelJuSo cells; n = 2.

To elucidate the unique mechanism of SQSTM1 function within the RNF26 protein network, we delved into the contribution of its UBA domain. Given previously reported connections between ubiquitin binding and ubiquitination of UBD-containing proteins (Sorkin, 2007), we considered whether SQSTM1 constitutes a substrate for RNF26. Indeed, major enhancement in short ubiquitin conjugates on SQSTM1 was observed in response to ectopic expression of wild-type but not catalytically inactive RNF26 relative to vector control (Figures 6A and 6B). Importantly, RNF26 was unable to ubiquitinate truncated SQSTM1 lacking its UBA domain (Figures 6A and 6B), indicating that only SQSTM1 in possession of its ubiquitin-binding faculties can serve as a substrate for the ER-located ubiquitin ligase. Taken together with the findings presented in Figure 5, the above evidence suggests that ubiquitinated SQSTM1 comprises the Ub-rich signals observed at sites of RNF26 activity.

**Figure S5:**
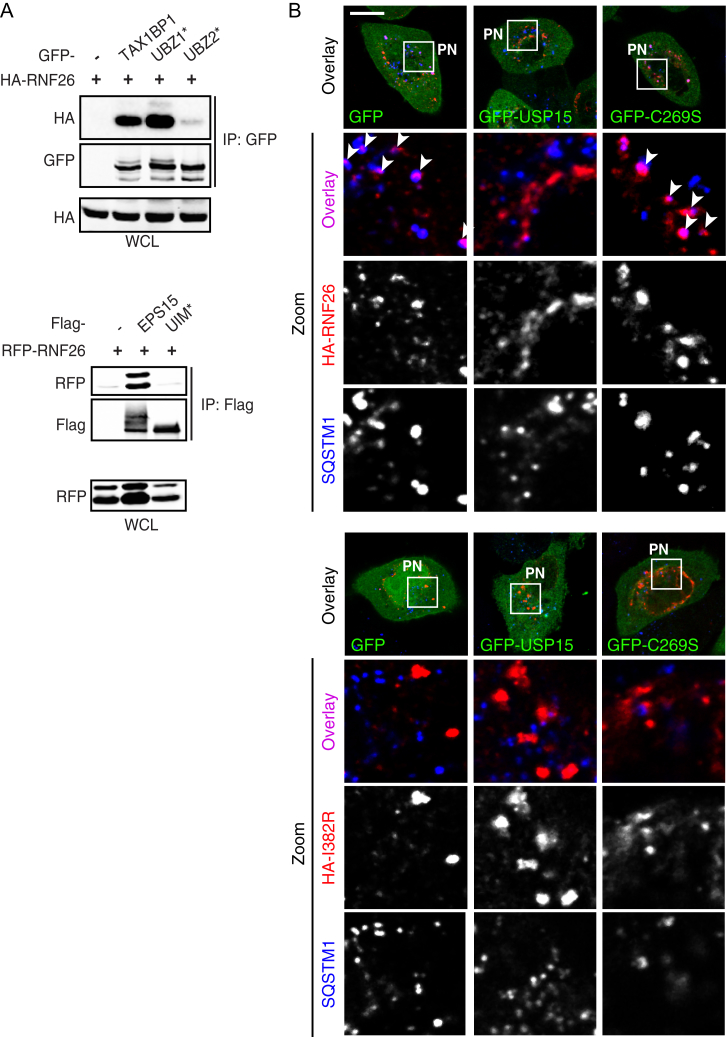
RNF26 Interacts with Ubiquitin-Binding Domains of Vesicle Adaptors, and USP15 Reduces Co-localization of RNF26 and SQSTM1, Related to Figures 5 and 6, Respectively (A) (Top panel) Complex formation between HA-RNF26 and either GFP-TAX1BP1 or its mutants UBZ^∗^ and UBZ2^∗^. (bottom panel) Complex formation between RFP-RNF26 and either FLAG-EPS15 or its mutant UIM^∗^. WCL: whole-cell lysate. For quantification of complex formation under the above and other conditions, see Figure 5D. (B) HeLa cells expressing active (top panel) or inactive (bottom panel) HA-RNF26 (red) along GFP-USP15 or its inactive mutant GFP-C269S (green) were stained for endogenous SQSTM1 (blue). A three-color overlay of a representative cell is shown in the top image for each condition and a zoom-in of the indicated perinuclear (PN) region for SQSTM1 and HA-RNF26 or its mutants is indicated below. Arrows point toward locations of colocalization of RNF26 and SQSTM1. Quantification appears in Figure 6D; n = 2, scale bar, 10 μm.

If ubiquitination afforded by RNF26 restricts vesicles in the PN cluster, a deubiquitinating activity may then complete the biochemical cycle to allow release of vesicles for rapid transit in the cell’s periphery. This function could be served by USP15, which preferentially associates with catalytically competent RNF26 (Figures 5A and 5D). We therefore tested whether USP15 deubiquitinates the RNF26 substrate, SQSTM1. Indeed, overexpression of wild-type USP15 dramatically decreased short-chain modification of SQSTM1 with ubiquitin in a manner dependent on its catalytic Cys 269 residue (Figures 6A and 6B). Furthermore, expression of wild-type (but not inactive) USP15 reduced the degree of colocalization of RNF26 with SQSTM1 (Figures 6C, 6D, and S5B), indicating that USP15 activity modulates occupancy of ligase-positive sites. Consistent with the notion that USP15 functionally rivals RNF26, silencing USP15 essentially ablated the highly mobile peripheral contingent marked by Lysotracker (Figure 6E; Movies S5A and S5C), resulting in an overall decrease in mobility of acidified organelles (Figure 6F)—a phenotype opposite to that observed with depletion of RNF26 (Figures 1E and 1F). As expected, based on their catalytic opposition, co-depletion of USP15 and RNF26 partially restored the PN/PP balance (Figure 6G), implying that USP15 promotes release of vesicles captured and restrained by the active RNF26 complex.

### The ER-Located RNF26/SQSTM1 Complex Controls Vesicle Positioning and Dynamics

**Figure S6:**
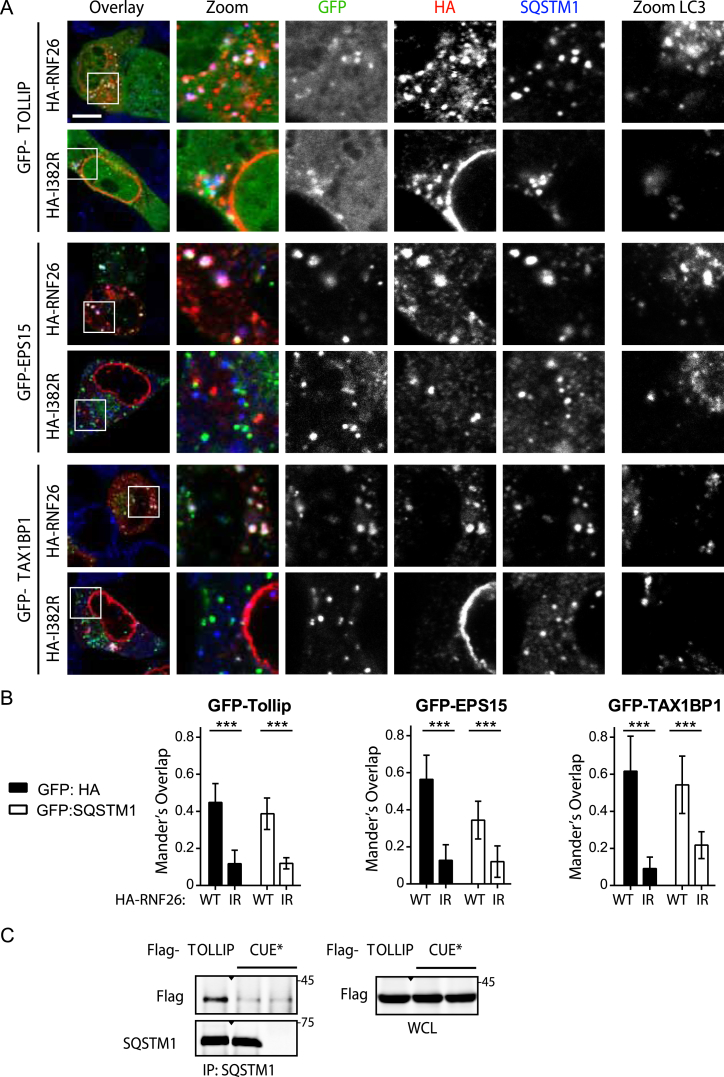
Vesicle Adaptors EPS15, TOLLIP, and TAX1BP1 Colocalize with SQSTM1 at RNF26-Positive Sites on the ER, Related to Figure 7 (A) Co-localization of GFP-TOLLIP (top panels), GFP-EPS15 (middle panels) or GFP-TAX1BP1 (bottom panels) with wild-type HA-RNF26 (red) versus catalytically inactive I382R (red) and SQSTM1 (blue) is shown as representative 3-color overlays and insets with their corresponding individual channels. Co-staining against LC3 is shown to the right. For dynamics of adaptor-selected vesicles associated with the RNF26/SQSTM1 complex see Figures 7 and S7. (B) Quantification of (A). Co-localization (Mander’s overlap) of either RNF26 (HA, black bars) or SQSTM1 (white bars) with GFP-TOLLIP (left), GFP-EPS15 (middle) or GFP-TAX1BP1 (right) is given for cells expressing the active (WT) or inactive (IR) form of RNF26. n = 2. (C) Complex formation between endogenous SQSTM1 and either FLAG-TOLLIP or its mutant CUE^∗^. WCL: Whole-cell lysate. Scale bar, 10 μm; n = # independent experiments; shown are the mean + error bars = SD.

Consistent with the proposed role of SQSTM1 in bridging adaptor-selected vesicles to catalytically competent RNF26, we observed colocalization of all three specific membrane adaptors—EPS15, TAX1BP1, and TOLLIP—with endogenous SQSTM1 at sites of wild-type, but not inactive RNF26 (Figures S6A and S6B). Additionally, the interaction between TOLLIP and SQSTM1 was exquisitely sensitive to mutation in the UBD domain of the former (Figure S6C), recapitulating ubiquitin-mediated recognition of RNF26 complexes by adaptor proteins (Figure 5). Importantly, structures positive for RNF26 and SQSTM1 did not overlap with the autophagy marker LC3 (Figure S6A, right panels), indicating that the function of SQSTM1 in this context is unrelated to autophagy.

**Figure 7:**
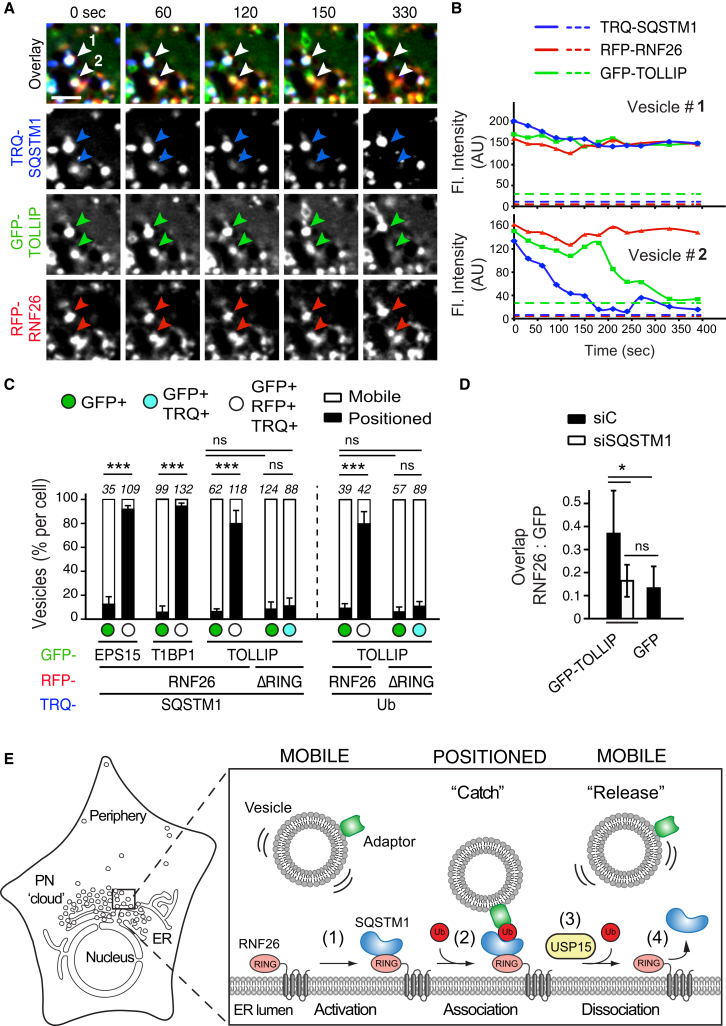
RNF26/SQSTM1 Complex Positions and Retains Adaptor-Selected Vesicles (A) Overlay zooms of frames selected from a time lapse (Movie S7) of vesicles marked by GFP-TOLLIP (green) in the presence of TRQ-SQSTM1 (blue) and RFP-RNF26 (red) in HeLa cells. Arrowheads point to two vesicles profiled in (B). Scale bar, 2.5 μm. (B) Plots of signal intensities over time corresponding to a positioned vesicle 1 (top graph) and a released vesicle 2 (bottom graph) as observed in (A). Dashed lines show background signal for each channel. (C) Quantification of adaptor-selected vesicle dynamics (mobile, white; positioned, black) expressed as % of vesicles per category (number counted given above each bar). GFP-marked vesicles (green); vesicles colocalizing with RFP-RNF26/-ΔRING and/or TRQ-SQSTM1 (white and cyan, respectively). See also Figures S6 and S7 and Movies S6A and S6B. (D) Co-localization (Mander’s overlap) between RNF26 and GFP-TOLLIP in control (siC) and SQSTM1-depleted (siSQSTM1) MelJuSo cells. (E) Model of vesicle positioning in the PN cloud by the RNF26 system. (1) Adaptor-selected (green) vesicles are subject to fast microtubule-based transport when unanchored by RNF26. (2) Catalytically competent RNF26 (light red) recruits SQSTM1 (blue) and mediates ubiquitin ligation (red), which serves to attract UBDs of specific vesicle-associated adaptors. On engagement, this multi-protein complex positions cognate vesicles (early, recycling, and late endosomes, and TGN) in the perinuclear space. (3) Dissociation of the RNF26/SQSTM1 complex, promoted by the DUB USP15 (yellow), releases target vesicles for (4) fast transport into the cell periphery.

**Figure S7:**
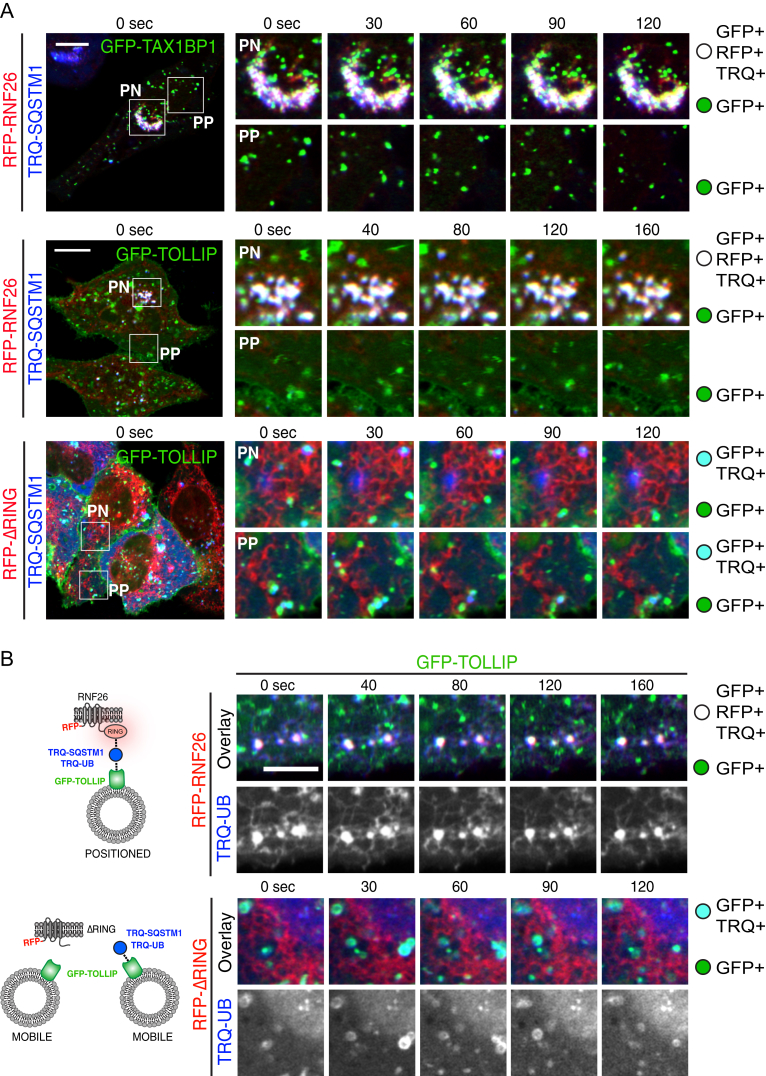
RNF26/SQSTM1 Complex Retains Adaptor-Selected Vesicles in the Perinuclear Cloud, Related to Figure 7 (A) Overlay confocal frame insets of selected perinuclear (PN) and peripheral (PP) regions from time lapses of HeLa cells co-expressing TRQ-SQSTM1 (blue) and GFP-TAX1BP1 (top, green), GFP-TOLLIP (middle and bottom, green) in the presence of either RFP-RNF26 (red) or RFP-ΔRING (red), as indicated. Quantification appears in Figure 7C. See also Movies S6A and S6B. (B) Schematic illustration of the suggested interaction between RNF26 and RNF26-ΔRING with SQSTM1 and the adaptors on vesicles. Colors correspond to the proteins as shown in (A). Snap shots of HeLa cells expressing RFP-RNF26 (top panels, red) or mutant RFP-RNF26ΔRING (bottom panel, red), TRQ-ubiquitin (blue) and GFP-TOLLIP (green). Three-color zoom-ins and Ubiquitin single (gray) images of a time lapse of the perinuclear region are shown. Quantification appears in Figure 7C. Scale bar, 10μm.

To test whether SQSTM1 dictates endosome positioning at RNF26, we monitored vesicle dynamics via specific membrane adaptors in living cells co-expressing fluorescent SQSTM1. We observed stable PN contacts between GFP-adaptors (“green”) and RFP-RNF26 (“red”) that were overwhelmingly positive for TRQ-SQSTM1 (“blue”; Figure S7A; Movie S6A), with tripartite complex formation (appearing “white” in the overlay) strongly correlated to fixed positional residence of vesicles marked by EPS15, TAX1BP1, and TOLLIP (Figures 7A–7C). By contrast, the vast majority of only GFP-positive “green” vesicles remained subject to fast transport (Figure 7C; Movie S6A). As expected, RNF26 lacking its RING domain could not mediate stable contacts with TRQ-SQSTM1 and failed to stabilize vesicles in position over time (Figures 7C and S7A; Movie S6B). Similar to TRQ-SQSTM1, TRQ-ubiquitin was found at contracts between GFP-TOLLIP-positive vesicles retained by RFP-RNF26 (but not RFP-ΔRING), while highly mobile vesicles were free of ligase and ubiquitin contacts (Figures S7B and 7C).

Interestingly, while most “white” vesicles stayed docked at the RNF26/SQSTM1 complex over time, occasional release was observed following disappearance of TRQ-SQSTM1 (Figures 7A and 7B, vesicles 1 and 2, respectively; Movie S7), and depletion of SQSTM1 markedly reduced distribution of TOLLIP-positive structures to RNF26 (Figure 7D). Considering diminished RNF26 occupancy by SQSTM1 in the presence of USP15 (Figures 6C and 6D), these findings suggest that assembly of SQSTM1 contacts at sites of RNF26 positions vesicles in the PN cloud, while disintegration of such complexes mediates vesicle release.

Taken together with the interaction and functional studies, the observations described above are consistent with the following order of molecular events: catalytically competent RNF26 recruits SQSTM1, which becomes subject to UBA-dependent ubiquitination by the ligase. This ubiquitin-rich RNF26/SQSTM1 complex is then poised to attract UBDs of endocytic adaptors (and the DUB USP15) to the PN cloud (proposed model depicted in Figure 7E). Subsequently, deubiquitination by USP15 at these sites determines release of the SQSTM1/Ub/Adaptor complex from the ER membrane, allowing transport of vesicles into the cell periphery). Collectively, our findings illustrate that by attracting diverse membrane-associated vesicle adaptors through a common mechanism, the RNF26/SQSTM1 complex controls the positioning and dynamics of endosomal vesicle transport and so designs the architecture of the endo- and exosomal system.

## Discussion

Proper control of the biosynthetic and endocytic membrane networks is crucial to normal functioning of cells and organisms, and failures therein are known to result in neuronal diseases (van der Kant and Neefjes, 2014) and obstructed immune responses (Watts, 2012), as well as contribute to a variety of cancers (Mellman and Yarden, 2013). The biology of endosomes (and the TGN) relies on cargo acquisition and vesicle transport working together to ensure accurate and timely delivery of select materials to their destinations. While we understand various aspects of cargo selection and vesicle transport, we know very little of the molecular decisions required to negotiate their arrivals and departures in the busy 3D environment of the cell. In the present study, we explored the functional relationship between the endosomal system’s architecture and dynamics, exposing its molecular underpinnings.

Cells organize a wide variety of their endosomal flavors (including vesicles of the TGN) into a PN cloud positioned near the MTOC. When in the cloud, vesicles exhibit restricted mobility, with only a fraction of each subtype traveling fast to and from the cell’s periphery at any given time. We show that the central player governing this spatiotemporal integrity—the ER-located E3 ligase RNF26—retains endosomes on location of its choice through post-translational modification with ubiquitin. Residing in the subdomain of the ER proximal to the nucleus, RNF26 draws the perinuclear architecture of the complex vesicle network suggestive of a cargo bazaar—a meeting place accessible from all corners of the cell, where efficient exchange can take place. We show that vesicles, internalized by either fluid-phase or ligand-mediated endocytosis, are targeted to the PN cloud, where they meet the late endosomal contingent. Loss of RNF26 function inhibits these encounters, slowing maturation of endosomes acquired in the periphery, without affecting the internalization rate. Furthermore, RNF26 depletion delays degradation of activated—i.e., signaling-competent—EGFR following ligand exposure, substantiating the notion that the spatiotemporal control afforded by the PN cloud facilitates vesicle maturation and cargo trafficking. Taken together with a recent report connecting a lysosome’s pH with its distance from the nucleus (Johnson et al., 2016), our findings argue that spatial information is intimately connected to function within the endosomal system.

To uncover the molecular mechanism responsible for PN cloud integrity, we identified RNF26-interacting proteins involved in the establishment and regulation of the PN cloud. Enabled by its catalytic activity, RNF26 employs ubiquitin-based communication with several membrane-associated ubiquitin-binding vesicle adaptors—TOLLIP (Ankem et al., 2011), EPS15 (Benmerah et al., 1999), and TAX1BP1 (Morriswood et al., 2007)—each exhibiting unique compartment selectivity. Importantly, all of the above adaptors in question are critical modulators of different signal transduction pathways. While EPS15 is phosphorylated by EGFR (Fazioli et al., 1993), TAX1BP1 binds TRAF6 downstream of TLRs and IL-1R (Ling and Goeddel, 2000), and TOLLIP interacts with TLR2/4 to inhibit innate immune response signaling (Zhang and Ghosh, 2002). Given that RNF26 silencing attenuates ligand-mediated receptor clearance by the endocytic pathway (as shown here for EGFR), the ER-located ubiquitin ligase may influence diverse signaling pathways emanating downstream of cell surface receptors when the latter are targeted by cognate adaptors to the PN cloud.

To signal vesicle recruitment, catalytically competent RNF26 attracts and ubiquitinates SQSTM1, which then serves as a platform for downstream ubiquitin/UBD-mediated complex assembly. These findings add a new dimension to the functional repertoire of SQSTM1, apart from its established role as an autophagic substrate adaptor (Lippai and Löw, 2014) and its recently reported involvement in dynein-mediated transport (Calderilla-Barbosa et al., 2014). Thus, through ubiquitin-based recognition, the ER controls the location and influences the dynamics of different vesicles with distinct biological functions. Given that TOLLIP, EPS15, and TAX1BP1 were isolated from a human melanoma cell line using a proteomic approach, it stands to reason that other vesicle-associated adaptor proteins expressed in other cell types could exhibit a similar relationship with RNF26. Indeed, the endocytic system is notoriously rich in UBD-containing adaptors (Raiborg and Stenmark, 2009, Shields et al., 2009), and some of these could, in principle, read positional signals from RNF26 as well.

If RNF26-associated machinery could only catch vesicles without being able to let go, the dynamic integrity of the system would be abolished. The breadth of ubiquitin-based recognition in dynamic biological processes (Pickart, 2001) is due in large part to its controlled reversibility, with DUB activities often functionally accompanying ubiquitin ligation (Clague et al., 2012). We show that RNF26 interacts with the DUB USP15, which influences occupancy of RNF26 by their common substrate, SQSTM1. Through their catalytic opposition, the two enzymes negotiate the delicate architectural/dynamic balance between the PN cloud and the periphery. How USP15 selects vesicles to be released is at present unclear, but may involve targeted localization or activation of its deubiquitinating functionality at specific RNF26-SQSTM1-adaptor complexes cleared for release.

It is becoming increasingly clear that the cell biology of endosomes and associated vesicle repertoires is modulated by their proximity to other membranes, and contacts with the ER feature prominently in this regard (Helle et al., 2013, Raiborg et al., 2015, Rocha et al., 2009). The sheer expansiveness of the ER provides a broadly available docking platform—an intracellular 3D grid—whereupon the RNF26 system could be uniquely capable of facilitating various aspects of vesicle biology. This is gleamed from our observations on delays in endosomal progression and cargo trafficking under conditions of PN cloud breakdown afforded by silencing RNF26. Furthermore, as a consequence of temporarily restricting vesicle mobility, the ER-located RNF26 could assist in the logistics of complex molecular processes, such as fission and/or fusion, both reportedly dependent on ER-endosome contact sites (Rowland et al., 2014, van der Kant and Neefjes, 2014). Our findings take a key step toward understanding how cells determine and manipulate the location of their highly mobile endosomal constituents and unveil a new facet of influence the ER exerts over the endosomal system.

## Experimental Procedures

Descriptions of cell lines, culture conditions, reagents, antibodies, and DNA constructs can be found in the Supplemental Experimental Procedures.

### siRNA Delivery

Silencing was performed as previously described (Paul et al., 2011) using siRNA oligos purchased from Dharmacon. For protocol and sequence details refer to the Supplemental Experimental Procedures.

### Light Microscopy

Samples were prepared as described in the Supplemental Experimental Procedures. Fixed and live samples were imaged using 63× lenses on Leica SP5 confocal microscopes adapted with a climate control chamber. To calculate fractional distances, fluorescent intensities along multiple line ROIs (assessed on maximum z projections using the line profile tool in LAS-AF software) were background corrected based on signal thresholds and normalized to median. Fractional distances were reported relative to the maximum distance from the center of the nucleus to the cell perimeter along a given trajectory. Vesicle tracking during time lapses was performed using TrackMate for Fiji. Fluid phase endocytosis was performed using SR101 as previously described (Wubbolts et al., 1996). Colocalization was reported as Mander’s coefficients calculated using JACoP for ImageJ. All error bars correspond to SD of the mean. Statistical evaluations report on Student’s t test (analysis of two groups) or one-way ANOVA analyses (analysis of three or more groups), with ^∗^p < 0.05, ^∗∗^p < 0.01, and ^∗∗∗^p < 0.001 (ns, not significant).

For additional details and descriptions of endocytosis and EGFR degradation assays, as well proteomic and biochemical methods, refer to the Supplemental Experimental Procedures.

## Author Contributions

M.J. and I.B. designed, conducted, and interpreted the majority of the experiments and prepared the manuscript. R.H.W. performed the experiments in Figures 3C and S3C and advised on data presentation throughout the manuscript. P.V. and G.J. performed mass spectrometry on prepared samples. L.J. and M.M. provided technical support. H.J. advised on endosome morphology. M.G. contributed to the study of TOLLIP function on late endosomes. R.S. discussed the results throughout the project. J.N. supervised the project. All authors edited the manuscript.

## Supplemental Information

Document S1. Supplemental Experimental Procedures

Movie S1. The Perinuclear Cloud of Late Endosomes Scatters with RNF26 Depletion, Related to Figure 1 3D compilations of (A) control MelJuSo cells (siC) or (B) those depleted of RNF26 (siRNF26_1). Overlays of endogenous CD63 (green) with nuclear DAPI (blue) are shown. See also Figure 1B.

Movie S2. Late Endosome Dynamics Are Coupled to the Overall Architecture of the Compartment, Related to Figure 1 (A–C) Time lapses of acidified vesicle (Lysotracker FarRed, white) dynamics as a function of RNF26 depletion in (A) control MelJuSo cells (siC) versus those depleted of RNF26 using two different siRNA duplexes (B siRNF26_1 and C siRNF26_2). Cell boundaries and nuclei are demarcated on the basis of a transmission image acquired at t = 0 sec. (3 sec between frames, 10 fps; scale bar, 10μm). (D and E) Time lapses of late endosome (mCherry-CD63, white) dynamics as a function of RNF26 depletion in (D) control MelJuSo cells (siC) and (E) those depleted of RNF26 (siRNF26_1) (3 sec between frames, 10 fps; scale bar, 10μm).

Movie S3. TGN Vesicles and Early Endosomes Are among the Perinuclear Cloud Members, Related to Figure 2 (A and B) Time lapses of TGN46-GFP in (A) control MelJuSo cells (siC) or (B) those depleted of RNF26 (siRNF26_1). Cell boundaries and nuclei are demarcated on the basis of a transmission image acquired at t = 0 sec. (7 sec between frames, 10 fps; scale bar, 10μm). (C and D) Time lapses of GFP-Rab14 in (C) control MelJuSo cells (siC) or (D) those depleted of RNF26 (siRNF26_1). Cell boundaries and nuclei are demarcated on the basis of a transmission image acquired at t = 0 sec. (7 sec between frames, 10 fps; scale bar, 10μm).

Movie S4. Endosomal Compartment Architecture Facilitates Maturation, Related to Figure 2 Time lapses of SR101 (green) trafficking to the acidified compartment (Lysotracker Green, magenta) in (A) control MelJuSo cells (siC) or (B) those depleted of RNF26 (siRNF26_1). SR101-only channel (white) and 2-color overlay are shown (2 min between frames, 15 fps; scale bar, 10μm).

Movie S5. Effects of Late Endosomal Adaptor TOLLIP on the Dynamics of the Endocytic Compartment, Related to Figures 4 and 6 Time lapses of acidified vesicle (Lysotracker FarRed, magenta) dynamics in (A) control HeLa cells (siC; cell boundaries and nuclei demarcated on the basis of a transmission image acquired at t = 0 sec; 3 sec between frames, 15 fps; scale bar, 10μm), (B) those ectopically expressing late endosomal adaptor GFP-TOLLIP (green; 7 sec between frames, 15 fps; scale bar, 10μm), or (C) cells depleted of DUB USP15 (siUSP15; 3 sec between frames, 15 fps; scale bar, 10μm).

Movie S6. Depletion of USP15 Abrogates the Peripheral Contingent of Late Endosomes, Related to Figures 7 and S7 (HeLa cells) Time lapses showing vesicle dynamics of GFP-TOLLIP (green) in the presence of TRQSQSTM1 (blue) and either (A) wild-type RFP-RNF26 (red; 40sec between frames) or (B) its mutant RFP-ΔRING (red; 30sec between frames) (5fps; scale bar, 10μm).

Movie S7. The RNF26/SQSTM1 Complex Positions Adaptor-Selected Endosomes, Related to Figure 7 (HeLa cells) Time lapse showing dynamics of GFP-TOLLIP (green) vesicles in the presence of RFPRNF26 (red) and TRQ-SQSTM1 (blue) in the perinuclear area. Three-channel overlay (white) along with single channels (white) are shown. Arrows point to vesicles 1 and 2, as described in Figure 7B (30 sec between frames, 2 fps; scale bar, 2.5μm)

Document S2. Article plus Supplemental Information
