# Supplementary material for: An ER-Associated Pathway Defines Endosomal Architecture for Controlled Cargo Transport
Source: Cell. 2016 Jun 30;166(1):152–66. doi: 10.1016/j.cell.2016.05.078 (PMC4930482; doi:10.1016/j.cell.2016.05.078)
Supplement: Document S1. Supplemental Experimental Procedures [file mmc1.pdf]

Cell, Volume *166*

Supplemental Information

An ER-Associated Pathway Defines Endosomal Architecture for Controlled Cargo Transport

Marlieke L.M. Jongsma, Ilana Berlin, Ruud H.M. Wijdeven, Lennert Janssen, George M.C. Janssen, Malgorzata A. Garstka, Hans Janssen, Mark Mensink, Peter A. van Veelen, Robbert M. Spaapen, and Jacques Neefjes

Extended Experimental Procedures

Cell Lines and Culturing: MelJuSo (human melanoma cell line), BJET (human fibroblast), HCT116 (human colon carcinoma) and RKO (human colon carcinoma) cells were cultured in IMDM (Gibco) supplemented with 7.5% fetal calf serum (FCS, Greiner). Human HEK293T cells and HeLa cells were cultured in DMEM (Gibco) supplemented with 7.5% fetal calf serum (FCS, Greiner). Monocytes were isolated from human blood using CD14 microbeads, and differentiated into macrophages (M-CSF, 50ng/ml, for 9 days) and immature dendritic cells (IL4 (800U/ml), GM-CSF (1000U/ml) for 6 days) by A. Thielen, A. Zaal and A. Saris (Sanquin Research). A MelJuSo cell line stably expressing TGN46-GFP was used in relevant live cell experiments.

Antibodies and fluorescent dyes: *(Confocal Microscopy)* Rabbit anti-HC (NKI), mouse anti-HC10 (NKI), rabbit anti-human HLA-DR (Neefjes et al., 1990), mouse anti-TrfR (Invitrogen 905963A), mouse anti-EEA1 (mAb 610457, BD transduction laboratories), mouse anti-CD63 NKI-C3 (Vennegoor and Rumke, 1986), mouse anti-clathrin (X22 CP45, Calbiochem), rabbit anti-TGN46 (Thermo Scientific, PA5-23568), rabbit anti-Giantin (Covance PRB-114C), mouse anti-M6PR (ab2733, Abcam), mouse anti-ubiquitin (mAb, P4D1, sc-8017, Santa Cruz), rabbit anti-LC3 (Novus Biologic), mouse anti-SQSTM1 (mAb, sc-28359, Santa Cruz), goat anti-VAP-A (Santa Cruz), and Rat anti-HA (3F10, Roche) were used to stain MHC class I, HLA-DR (Late endosomes/MVBs), EEA1 (Early endosomes), CD63 (Late endosomes), clathrin, TGN46 (Trans Golgi Network), Giantin (Golgi), M6PR (secretory vesicles), ubiquitin, LC3 (autophagosomes), SQSTM1, VAP-A and HA-tagged proteins respectively, followed by secondary anti-Rabbit/Mouse/Rat Alexa-dye coupled antibodies (Invitrogen) for detection by confocal microscopy. DAPI (Sigma), Lysotracker deep red (Life Technologies) and Lysotracker green (DND-260, Molecular probes; 0.1µM added for 15-30min before fixation) were used to stain the nucleus and lysosomes for detection by confocal microscopy. Sulforhodamine 101 (SR101, Sigma), 25µg/ml; Wubbolts et al, 1996) and EGF-Alexa555 (100ng/ml, Invitrogen) were used in endocytosis assays. *(Flow cytometry)* anti-HLA-DR (APC) (347403, BD), EGFR (Alexa647) (528, sc-120 Santa Cruz) and anti-CD63 (NKI-C3) (DyLight647 conjugated, Thermo Scientific) were used to stain MHC class II, EGFR and CD63 at the cell surface. *(Western Blotting)* mouse anti TfR (905963A, Invitrogen), rabbit anti-EGFR (Millipore), mouse anti-phosphotyrosine (pY; 4G10 Millipore), mouse anti-HLA DRα (1B5, NKI), mouse anti-SQSTM1 (mAb, sc-28359, Santa Cruz), rabbit anti-Rab9A (NKI), mouse anti-LAMP1 (BD, 611042), rabbit anti-USP15 (Bethyl, A300-923A), goat anti-TOLLIP (sc27315, Santa Cruz), rabbit anti-TAX1BP1 (A303-791A, Bethyl), rabbit anti-EPS15 (pAb, sc-1840, Santa Cruz), rabbit anti-mGFP (Rocha et al., 2009), mouse anti-HA (HA.11 (16B12), Covance MMS-101R), anti-mRFP (Rocha et al., 2009), rabbit anti-FLAG (F7425, Sigma), mouse anti-FLAG M2 (F3165, Sigma) and mouse anti B-actin (AC-

15, Sigma) followed by secondary Rabbit anti-Mouse-PO (P0161, Dako) or HRP-Protein A (10-1023, Invitrogen) were used for detection of endogenous or overexpressed proteins by Western blot. Secondary IRDye 680LT Donkey anti-Goat IgG (H+L) (926-68024, Li-Cor), IRDye 800CW Goat anti-rabbit IgG (H+L) (926-32211, Li-COR), IRDye 800CW Goat anti-mouse IgG (H+L) (926-32210, Li-COR), IRDye 680LT Goat anti-rabbit IgG (H+L) (926-68021, Li-COR) and IRDye 680LT Goat anti-mouse IgG (H+L) (926-68020, Li-COR) were used for detection using the Odyssey Classic imager (Li-Cor). *(Immuno-precipitation)* rabbit anti-Tollip (Sigma), rabbit anti-mGFP (Rocha et al., 2009), rabbit anti-FLAG (F7425, Sigma), rabbit anti-TAX1BP1 (A303-791A, Bethyl), rabbit anti-USP15 (Bethyl, A300-923A), mouse anti-SQSTM1 (mAb, sc-28359, Santa Cruz) and rabbit anti-EPS15 (pAb, sc-1840, Santa Cruz) were used for immuno-precipitation of tagged or endogenous proteins.

Constructs: RNF26 was amplified from IMAGE: 3507662 and cloned into mRFP-C1, mGFP-C1 and 2HA-C1 vectors by EcoRI/BamH1 restriction sites. Inactive mutants of RNF26 (I382R and C401S) were created by site directed mutagenesis. RNF26 ΔTM (aa 246-433) and RNF26 ΔRING (aa1-352) was amplified from the full length construct and cloned into mRFP-C1, mGFP-C1 and/or 2HA-C1 via EcoRI/BamHI Restriction sites. GST-RNF26 ΔTM (aa246-433) and GST-RNF26 RING (aa363-433) were cloned into pRP265 using BamHI/HindIII restriction sites and used for protein production. TOLLIP was cloned into mGFP-C1 and 2xFLAG-C1 by KpnI/HindIII restriction sites. GFP- and FLAG-TOLLIP M240A/F241A (CUE*) was created from the full-length construct by site directed mutagenesis. GFP-SQSTM1 and GFP-SQSTM1 ΔUBA (aa1-401) were amplified from Addgene plasmid #28027 and cloned into mGFP-C1 or mTurqoise-C1 (Goedhart et al., 2012) using EcoRI/XhoI restriction sites. GFP-SQSTM1 P392L (UBA*) was made from the full-length construct by site directed mutagenesis. TAX1BP1 (IMAGE: 100001732) was cloned into mGFP-C1 using the restriction enzymes Asp718 and BamHI, GFP-TAX1BP1 F737A (UBZ1*) and F764A (UBZ2*) were made from the full-length construct by site directed mutagenesis. GFP-EPS15, FLAG-EPS15 and FLAG-EPS15 L883A/L885A (UIM*) in pMT2SM were a gift from J. Borst (NKI, Amsterdam). USP15 (Addgene plamid #22570) was cloned into mRFP-C1 and mGFP-C1 using the restriction enzymes HinDIII and MluI. The inactive mutant USP15 C269A was generated from the full-length construct by site-directed mutagenesis. HA-ubiquitin in pcDNA3.1 was a generous gift from I. Dikic (Institute for Biochemie II, Frankfurt). mTurquoise-Ub was cloned into mTurquoise-C1 (Goedhart et al., 2012) using HindIII/Asp718I restriction sites. TGN46-GFP was amplified from IMAGE: 53117823 and cloned into mGFP-N1 by EcoRI/BamH1 restriction sites. GFP-Rab14 was cloned into eGFP-C1 (Kuijl et al., 2013). GFP-Rab7 was described before (I. Jordens et al., 2001). GFP-Rab5 was previously described (Jordens et al., 2001). All constructs were sequence verified.

Site directed Mutagenesis: Forward en reverse primers containing the desired mutations were created. A mixture containing template DNA, 1x Pfu buffer, 20mM dNTPs, 0.6µM forward primer, 0.6µM reverse primer, 1µl Turbo Pfu Polymerase filled to 50µl with DEPC was amplified using the following program: 95°C 2min; (95°C 30s; 52°C 30s; 68°C 13min + 2min/Kb) x 20 cycles; 68°C 20min; 4°C forever. 20µl amplified product was incubated with 2µl DpnI (Thermo scientific) for 4hrs at 37°C to digest the template DNA. The mutated DNA was transformed into DH5α using 2xYT medium to increase the amount of the mutated constructs.

siRNA transfection: Sequences of the siRNA oligos targeting RNF26 used in this study are given in the table below. In all cases siRNF26_1 was used unless otherwise indicated. For rescue experiments, siRNF26 3'UTR was used. Gene silencing was performed in a 24 well plate using 50µl siRNA (500nM stock) mixed with 0.75µl DharmaFECT1 #1 (Dharmacon) diluted in 49.25µl IMDM. Transfections performed in other dish/well volumes were scaled up according to media volume appropriate for standard culturing. In case of double or triple siRNA transfections a final concentration of 50nM in a 1:1 (or 1:1:1) ratio of the different siRNA duplexes was used. The mixture was incubated for 20min on a shaker followed by the addition of 28,000 MelJuSo cells in IMDM and cultured for three days at 37°C and 5%CO2 before analysis. Non-targeting siRNA (siCTRL, D-001206-13-20, Dharmacon) was used as a negative control. RNF26 interacting proteins EPS15, TAX1BP1, TOLLIP, SQSTM1 and USP15 were silenced using siRNAs from the siGenome SMARTpool library (Dharmacon).

**Gene**	**siRNA sequence (sense)**
RNF26_1 (siGENOME D-007060-17)	GAGAGGAUGUCAUGCGGCU
RNF26_2 (siGENOME D-007060-04)	GCAGAUCAGAGGCAGAAGA
RNF26 3'UTR (Custom, Thermo Sci)	CAGGAGGGAUAACCGGAUUUU

DNA transfections: MelJuSo, HeLa and RKO cells seeded in a 12-well plate were transfected using Effectine (Qiagen, 301427), according to manufacturer's protocol or using Extremegene HP (Roche): 100µl IMDM medium was mixed with 3µl Extremegene HP and 1µg DNA. After 30min, the mix was added to the MelJuSo cells and cultured for one day at 37°C and 5% CO2 before analysis. HEK293T cells seeded in a 6-well plate were transfected using PEI (Polyethylenimine, 23966, Polysciences Inc.). 100µl IMDM medium was mixed with 6µl PEI and

2µg DNA. After 30min, the mix was added to the HEK293T cells and cultured for one day at 37°C and 5% CO_2 before analysis.

EGFR degradation: Ligand-mediated turnover of EGFR was assayed as previously described (Berlin et al, 2010) using 20ng/ml EGF. Receptor abundance at each indicated time-point following stimulation was quantified relative to Transferrin receptor and expressed as a fraction of EGFR at t=0 for each condition. Receptor phosphorylation was expressed relative to the maximal activation achieved in control cells (siC).

Ubiquitination: HEK293T cells were lysed for 30 min in 0.5%TX100 lysis buffer containing 50mM Tris-HCl pH7.5, 150mM NaCl, 5mM EDTA, 0.5%TX100, freshly added 10mM NMM (DUB inhibitor diluted in DMSO) and protease inhibitors (Roche Diagnostics, EDTA free). Supernatants were frozen at -80°C, thawed and sonicated (Branson Sonifier 250, 3 pulses, Duty Cycle=50%, Output=7). After spinning (10 min at 12,000g), we incubated the lysates with antibody-coupled Protein G4 fast flow (GE Healthcare) for one hour. Beads were washed four times in 0.5%TX100 containing lysis buffer before addition of Laemmli Sample Buffer (containing 5% β-mercaptoethanol) followed by 5 min incubation at 95°C. Proteins were separated by SDS-PAGE (8% acrylamide gel), transferred to nitrocellulose membranes and detected by antibodies. Li-Cor fluorescent dyes were used as secondary antibodies and detected by an Odyssey Classic imager (Li-Cor).

Confocal Microscopy: For fixed samples, cells were fixed in PBS/3.75% formaldehyde (acid-free, Merck), permeabilized with PBS/0.1% TritonX-100 (T8787, Sigma) and blocked with PBS/0.5% (v/v) bovine serum albumin (BSA, A8022, Sigma) or PBS/5% (w/v) milk powder (Skim milk powder, LP0031, Oxiod). Cell were stained using desired antibodies diluted in PBS/0.5% bovine serum albumin or PBS/5% milk. Cells were mounted using ProLong Gold antifade Mountant with DAPI (P36941 Life Technologies) or Vectashield (Vector Laboratories). Samples were imaged using a Leica SP5 microscope with appropriate filters for fluorescence detection. Pictures were taken using a HCX PL 63x 1.32 oil objective and 1-4 digital zoom as applicable. Hoechst was excited at λ=405nm and detected at λ=416-470nm; Alexa-488 was excited at λ=488nm and detected at λ=500-550nm. Alexa-568 was excited at λ=561nm and detected at λ=570-621 nm; Alexa-647 was excited at λ=633nm and detected at λ=642-742nm. Z-stacks were imaged with a z-step size of 1.0µm and visualized as max z-projection and 90° using the LAS-AF images software 3D projection tool and the orthogonal sectioning respectively.

Quantification Confocal images: To calculate fractional distances fluorescent intensities along multiple line ROI (assessed by using the line profile tool of LAS-AF) were normalized to median

and background pixels were excluded from the analysis by determining the signal-treshold. Distances corresponding to the remaining (vesicular) pixels relative to the maximum distance were plotted as fractional distance in a dot-plot graph. Co-localization was quantified using ImageJ (LOCI and JACoP plug-in) determining the Manders coefficient (displayed as protein A: protein B = amount of protein A overlapping with protein B). Life cell imaging analyses were performed using a Leica SP5 microscope. Vesicle tracking was performed using TrackMate for Fiji (Vesicle diameter = 1µm; thresholds and other parameters were chosen as appropriate based on control samples within each experiment). Cell Profiler (2.1.1) was used for automated image analysis using DAPI and RaHC as nuclear and cytosolic markers respectively. For statistical analysis, p-values were determined using Student's t test or one-way ANOVA.

Flow cytometry: siRNA transfected MelJuSo and HeLa cells were washed in PBS, detached using Trypsin-EDTA 0.1% (Gibco) and incubated with fluorescent-conjugated antibodies recognizing MHC class II, EGFR and CD63 in FACS buffer (2%FCS/PBS) for 30min at 4°C. Internalization assays were performed by incubating cells with SR101 at the same concentration as used for imaging studies (see above) for up to 2 hours (taking time-points as indicated) at 37°C. Cells were washed in PBS and fixed in PBS containing 1% Formaldehyde (Merck). The Mean Fluorescent Intensity (MFI) of the stained cells was measured using a BD LSR Fortessa analyser.

qPCR: Messenger RNA was extracted from cells using the mRNA Capture Kit (11787896001, Roche) and reverse transcribed into cDNA using the Transcriptor High Fidelity cDNA Synthesis Kit (05081866001, Roche). Quantitative RT-PCR was performed using LightCycler® 480 SYBR Green 1 Master (04707516001, Roche) on the LightCycler® 480 Detection System (Roche). Primer sequences are listed in a table below. Quantification was performed using the comparative CT method (ΔΔCT). The results were expressed relative to 18S values; normalized to control siRNA treated cells and LOG-transformed.

**Gene**	**Primer sequence**
18S	(5'-3') CGGCTACCACATCCAAGGAA
	(3'-5') GCTGGAATTACCGCGGCT
RNF26	(5'-3') TCGGCACTCAGAACCTCTTT
	(3'-5') CTAGGAAGGCAGCCACTACG

GST-pulldown: 100 x10^6 MelJuSo cells were lysed for 30 min in 20ml lysis buffer containing 0.8% NP-40 (74385, Sigma), 50mM NaCl, 50mM Tris-HCl pH8.0, 5mM MgCl2, 10% Glycerol, 1mM DTT and phosphatase inhibitors (Roche Diagnostics, EDTA free). Nuclei and aggregates were removed (10min at max. speed) and the resulting supernatant was incubated with GST- or GST-RNF26 Tail (either aa304-433 or aa363-433), coupled Glutathione-Sepharose beads 4B (GE Healthcare) for 1hr (20µg protein/50µl beads). Beads were washed four times in Wash buffer (0.08% NP-40, 250mM NaCl, 50mM Tris-HCl pH 8.0 and 5mM MgCl2) before addition of Laemmli Sample Buffer (containing 5% β-mercaptoethanol) followed by 5min incubation at 95°C. Samples were separated by 4-12% SDS-PAGE (NuPAGE Bis-Tris Precast Gel, Life Technologies) and stained with silver (SilverQuest Silver Stain, Life Technologies).

Mass spectrometry: Selected bands (and the same region in the GST control lane as negative controls) were cut from the silver stained gel and subjected to reduction with dithiothreitol, alkylation with iodoacetamide and in-gel trypsin digestion using a Proteineer DP digestion robot (Bruker). Tryptic peptides were extracted from the gel, lyophilized, dissolved in 95/3/0.1 v/v/v water/acetonitril/formic acid and subsequently analyzed by on-line nanoHPLC MS/MS using an 1100 HPLC system (Agilent Technologies), as previously described (Meiring et al., 2002). Peptides were trapped at 10 µl/min on a 15-mm column (100-µm ID; ReproSil-Pur C18-AQ, 3 µm, Dr. Maisch GmbH) and eluted to a 200 mm column (50-µm ID; ReproSil-Pur C18-AQ, 3 µm) at 150 nl/min. All columns were packed in house. The column was developed with a 30-min gradient from 0 to 50% acetonitrile in 0.1% formic acid. The end of the nanoLC column was drawn to a tip (5-µm ID), from which the eluent was sprayed into a 7-tesla LTQ-FT Ultra mass spectrometer (Thermo Electron). The mass spectrometer was operated in data-dependent mode, automatically switching between MS and MS/MS acquisition. Full scan MS spectra were acquired in the FT-ICR with a resolution of 25,000 at a target value of 3,000,000. The two most intense ions were then isolated for accurate mass measurements by a selected ion-monitoring scan in FT-ICR with a resolution of 50,000 at a target accumulation value of 50,000. Selected ions were fragmented in the linear ion trap using collision-induced dissociation at a target value of 10,000. In a post-analysis process, raw data were first converted to peak lists using Bioworks Browser software v3.2 (Thermo Electron), and then submitted to the Swissprot database version 51.6 (257,964 entries), using Mascot v. 2.2.04 (www.matrixscience.com) for protein identification. Mascot searches were with 2 ppm and 0.8 Da deviation for precursor and fragment mass, respectively, and trypsin as enzyme. Protein was finally sorted and compared using Scaffold software version 3.0.1 (www. proteomesoftware.com).

Co-immunoprecipitation: HEK293T cells were lysed for 30min in lysis buffer containing 0.8% NP-40, 50 mM NaCl, 50 mM Tris-HCl pH8.0, 5mM MgCl2 and protease inhibitors (Roche

Diagnostics, EDTA free). Supernatant after spinning (10min at 12,000g) was incubated with antibody-coupled Protein G 4 fast flow (GE Healthcare) for 1hr. Beads were washed four times in Wash buffer containing 0.08% NP-40, 150mM NaCl, 50mM Tris-HCl pH 8.0 and 5mM MgCl2 before addition of Laemmli Sample Buffer (containing 5% β-mercaptoethanol) followed by 5min incubation at 95°C. Co-immunoprecipitated proteins were separated by SDS-PAGE for Western blotting and detection by antibody staining. Depending on the secondary antibodies used, antibody signals were detected by Chemidoc XRS+ imager (Bio-Rad) or Odyssey imager.

SDS-PAGE and Western blotting: Samples were separated by a 10% acrylamide gel and transferred to a nitrocellulose membrane (Protan BA85, 0.45µm, GE Healthcare) or PVDF membrane (Immobilon-P, 0.45µm, Millipore) at 300mA for 2hrs. The filters were blocked in PBS/0.1%Tween20 (P1379, Sigma-Aldrich)/5% Milk (Skim milk powder, LP0031, Oxiod) (nitrocellulose membranes used for Odyssey read-outs were blocked in PBS/5%Milk without Tween to reduce background) and incubated with a primary antibody for 1hr diluted in PBS/0.1%Tween/5% Milk, washed thrice for 10min in PBS/0.1% Tween and incubated with the secondary antibody for 45min diluted in PBS/0.1%Tween/5% Milk and washed thrice again in PBS/0.1% Tween. Depending on the secondary antibody, the filter was incubated with ECL reagent (SuperSignal West Dura Extended Duration Substrate, Thermo Scientific) and the signal was detected using the Chemidoc XRS+ imager (Bio-Rad) or directly imaged by the Odyssey Classic imager (Li-Cor). Intensity of bands was quantified using ImageLab or Image studio Software.

Supplemental references

Berlin, I., Schwartz, H., and Nash, P.D. (2010) Regulation of epidermal growth factor receptor ubiquitination and trafficking by the USP8/STAM complex. JBiol Chem *285(45)*, 34909-21.

Goedhart, J., von Stetten, D., Noirclerc-Savoye, M., Lelimousin, M., Joosen, L., Hink, M.A., van Weeren, L., Gadella, T.W., Jr., and Royant, A. (2012). Structure-guided evolution of cyan fluorescent proteins towards a quantum yield of 93%. Nature communications *3*, 751.

Jordens, I., Fernandez-Borja, M., Marsman, M., Dusseljee, S., Janssen, L., Calafat, J., Janssen, H., Wubbolts, R., and Neefjes, J. (2001). The Rab7 effector protein RILP controls lysosomal transport by inducing the recruitment of dynein-dynactin motors. Current biology : CB *11*, 1680-1685.

Kuijl, C., Pilli, M., Alahari, S.K., Janssen, H., Khoo, P.S., Ervin, K.E., Calero, M., Jonnalagadda, S., Scheller, R.H., Neefjes, J., *et al.* (2013). Rac and Rab GTPases dual effector Nischarin regulates vesicle maturation to facilitate survival of intracellular bacteria. The EMBO journal *32*, 713-727.

Neefjes, J.J., Stollorz, V., Peters, P.J., Geuze, H.J., and Ploegh, H.L. (1990). The biosynthetic pathway of MHC class II but not class I molecules intersects the endocytic route. Cell *61*, 171-183.

Rocha, N., Kuijl, C., van der Kant, R., Janssen, L., Houben, D., Janssen, H., Zwart, W., and Neefjes, J. (2009). Cholesterol sensor ORP1L contacts the ER protein VAP to control Rab7-RILP-p150 Glued and late endosome positioning. JCell Biol *185*, 1209-1225.

Vennegoor, C., and Rumke, P. (1986). Circulating melanoma-associated antigen detected by monoclonal antibody NKI/C-3. Cancer immunology, immunotherapy : CII *23*, 93-100.

Wubbolts, R., Fernandez-Borja, M., Oomen, L., Verwoerd, D., Janssen, H., Calafat, J., Tupl, A., Dusseljee, S., and Neefjes, J. (1996). Direct vesicular transport of MHC class II molecules from lysosomal structures to the cell surface. JCell Biol *135*, 611-622.
