# Supplementary figures and images for: An ER-Associated Pathway Defines Endosomal Architecture for Controlled Cargo Transport
Source: Cell. 2016 Jun 30;166(1):152–66. doi: 10.1016/j.cell.2016.05.078 (PMC4930482; doi:10.1016/j.cell.2016.05.078)

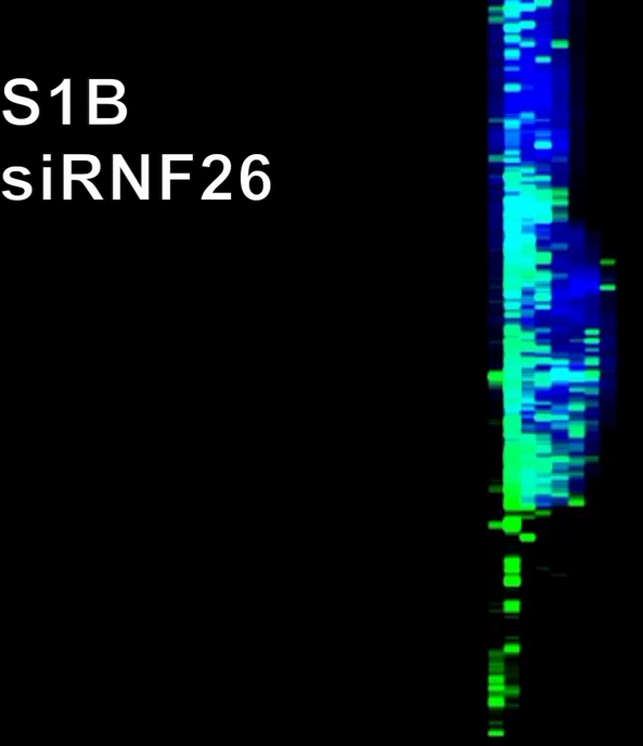

Supplement: Movie S1. The Perinuclear Cloud of Late Endosomes Scatters with RNF26 Depletion, Related to Figure 1 — 3D compilations of (A) control MelJuSo cells (siC) or (B) those depleted of RNF26 (siRNF26_1). Overlays of endogenous CD63 (green) with nuclear DAPI (blue) are shown. See also Figure 1B. [file mmc2.jpg]

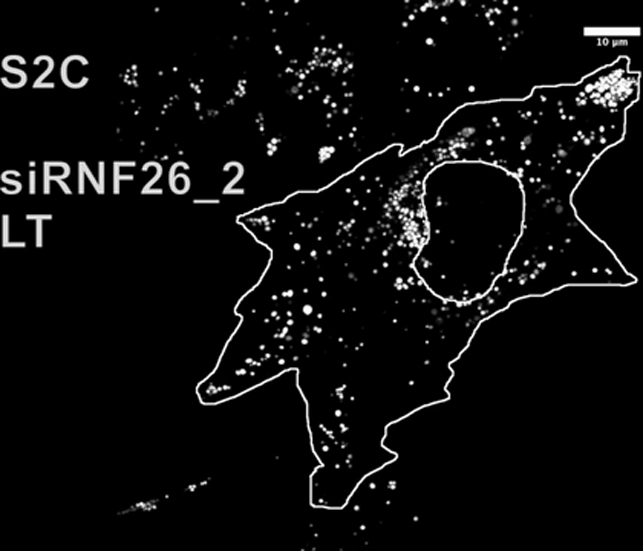

Supplement: Movie S2. Late Endosome Dynamics Are Coupled to the Overall Architecture of the Compartment, Related to Figure 1 — (A–C) Time lapses of acidified vesicle (Lysotracker FarRed, white) dynamics as a function of RNF26 depletion in (A) control MelJuSo cells (siC) versus those depleted of RNF26 using two different siRNA duplexes (B siRNF26_1 and C siRNF26_2). Cell boundaries and nuclei are demarcated on the basis of a transmission image acquired at t = 0 sec. (3 sec between frames, 10 fps; scale bar, 10μm). (D and E) Time lapses of late endosome (mCherry-CD63, white) dynamics as a function of RNF26 depletion in (D) control MelJuSo cells (siC) and (E) those depleted of RNF26 (siRNF26_1) (3 sec between frames, 10 fps; scale bar, 10μm). [file mmc3.jpg]

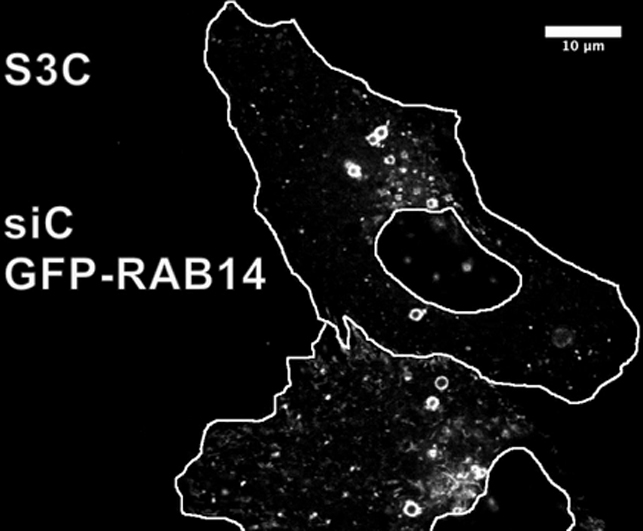

Supplement: Movie S3. TGN Vesicles and Early Endosomes Are among the Perinuclear Cloud Members, Related to Figure 2 — (A and B) Time lapses of TGN46-GFP in (A) control MelJuSo cells (siC) or (B) those depleted of RNF26 (siRNF26_1). Cell boundaries and nuclei are demarcated on the basis of a transmission image acquired at t = 0 sec. (7 sec between frames, 10 fps; scale bar, 10μm). (C and D) Time lapses of GFP-Rab14 in (C) control MelJuSo cells (siC) or (D) those depleted of RNF26 (siRNF26_1). Cell boundaries and nuclei are demarcated on the basis of a transmission image acquired at t = 0 sec. (7 sec between frames, 10 fps; scale bar, 10μm). [file mmc4.jpg]

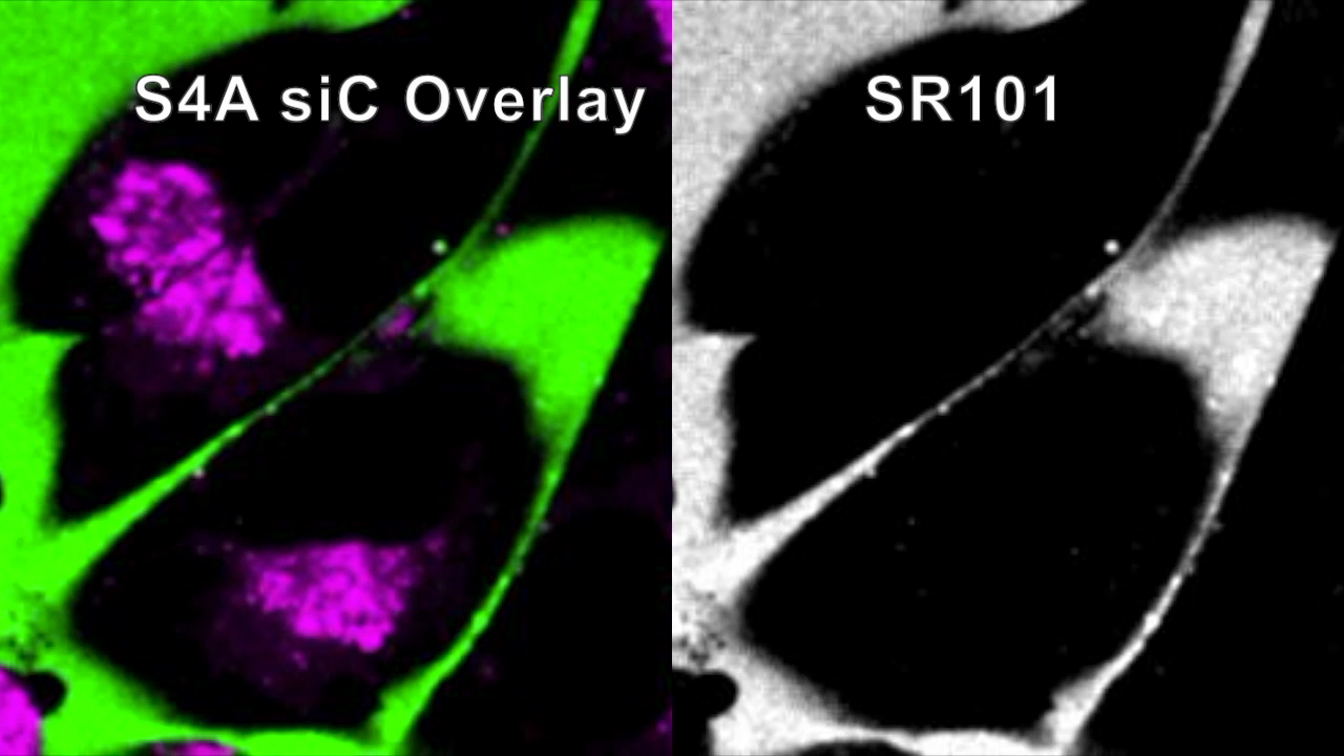

Supplement: Movie S4. Endosomal Compartment Architecture Facilitates Maturation, Related to Figure 2 — Time lapses of SR101 (green) trafficking to the acidified compartment (Lysotracker Green, magenta) in (A) control MelJuSo cells (siC) or (B) those depleted of RNF26 (siRNF26_1). SR101-only channel (white) and 2-color overlay are shown (2 min between frames, 15 fps; scale bar, 10μm). [file mmc5.jpg]

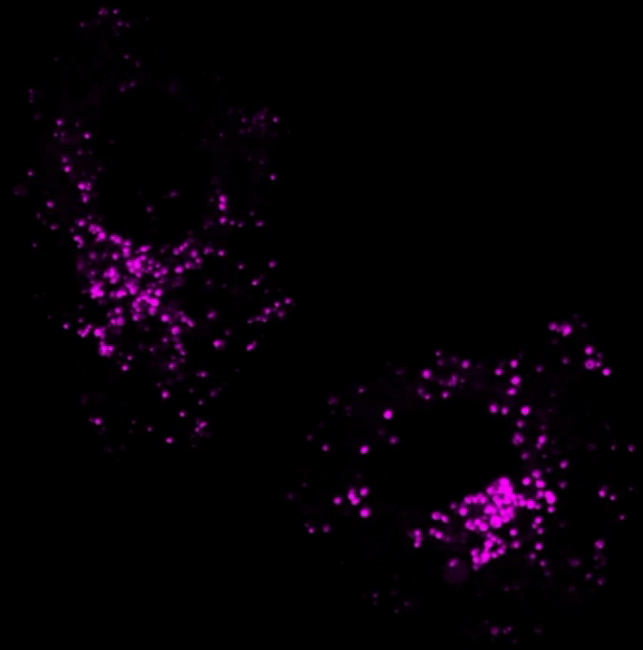

Supplement: Movie S5. Effects of Late Endosomal Adaptor TOLLIP on the Dynamics of the Endocytic Compartment, Related to Figures 4 and 6 — Time lapses of acidified vesicle (Lysotracker FarRed, magenta) dynamics in (A) control HeLa cells (siC; cell boundaries and nuclei demarcated on the basis of a transmission image acquired at t = 0 sec; 3 sec between frames, 15 fps; scale bar, 10μm), (B) those ectopically expressing late endosomal adaptor GFP-TOLLIP (green; 7 sec between frames, 15 fps; scale bar, 10μm), or (C) cells depleted of DUB USP15 (siUSP15; 3 sec between frames, 15 fps; scale bar, 10μm). [file mmc6.jpg]

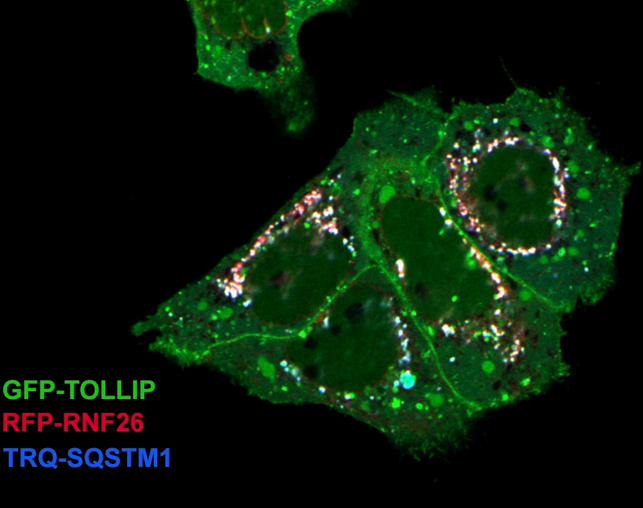

Supplement: Movie S6. Depletion of USP15 Abrogates the Peripheral Contingent of Late Endosomes, Related to Figures 7 and S7 — (HeLa cells) Time lapses showing vesicle dynamics of GFP-TOLLIP (green) in the presence of TRQSQSTM1 (blue) and either (A) wild-type RFP-RNF26 (red; 40sec between frames) or (B) its mutant RFP-ΔRING (red; 30sec between frames) (5fps; scale bar, 10μm). [file mmc7.jpg]

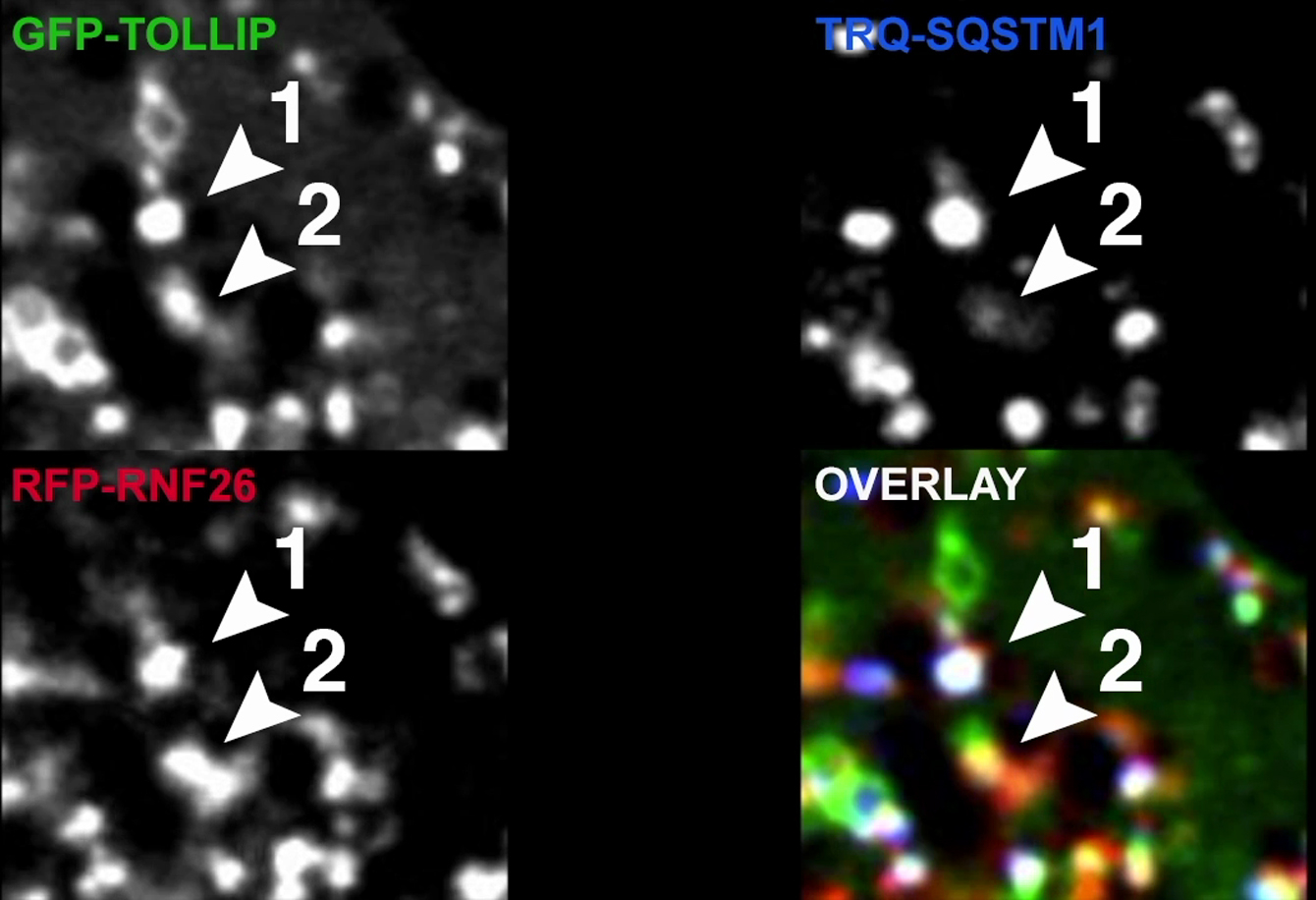

Supplement: Movie S7. The RNF26/SQSTM1 Complex Positions Adaptor-Selected Endosomes, Related to Figure 7 — (HeLa cells) Time lapse showing dynamics of GFP-TOLLIP (green) vesicles in the presence of RFPRNF26 (red) and TRQ-SQSTM1 (blue) in the perinuclear area. Three-channel overlay (white) along with single channels (white) are shown. Arrows point to vesicles 1 and 2, as described in Figure 7B (30 sec between frames, 2 fps; scale bar, 2.5μm) [file mmc8.jpg]
